# Mechanical cell competition kills cells via induction of lethal p53 levels

**DOI:** 10.1038/ncomms11373

**Published:** 2016-04-25

**Authors:** Laura Wagstaff, Maja Goschorska, Kasia Kozyrska, Guillaume Duclos, Iwo Kucinski, Anatole Chessel, Lea Hampton-O'Neil, Charles R. Bradshaw, George E. Allen, Emma L. Rawlins, Pascal Silberzan, Rafael E. Carazo Salas, Eugenia Piddini

**Affiliations:** 1 The Wellcome Trust/Cancer Research UK Gurdon Institute and Zoology Department, University of Cambridge, Tennis Court Road, Cambridge CB2 1QN, UK; 2 Laboratoire PhysicoChimie Curie, Institut Curie, Paris Sciences et Lettres Research University – Sorbonne Universités, Université Pierre et Marie Curie – Centre National de la recherche Scientifique – Equipe labellisée Ligue Contre le Cancer, 75005 Paris, France; 3 Pharmacology Department, University of Cambridge, Tennis Court Road, Cambridge CB2 1PD, UK

## Abstract

Cell competition is a quality control mechanism that eliminates unfit cells. How cells compete is poorly understood, but it is generally accepted that molecular exchange between cells signals elimination of unfit cells. Here we report an orthogonal mechanism of cell competition, whereby cells compete through mechanical insults. We show that MDCK cells silenced for the polarity gene *scribble* (*scrib*^*KD*^) are hypersensitive to compaction, that interaction with wild-type cells causes their compaction and that crowding is sufficient for *scrib*^*KD*^ cell elimination. Importantly, we show that elevation of the tumour suppressor p53 is necessary and sufficient for crowding hypersensitivity. Compaction, via activation of Rho-associated kinase (ROCK) and the stress kinase p38, leads to further p53 elevation, causing cell death. Thus, in addition to molecules, cells use mechanical means to compete. Given the involvement of p53, compaction hypersensitivity may be widespread among damaged cells and offers an additional route to eliminate unfit cells.

## Abstract (web summary)

Cell competition is a quality control mechanism to eliminate unfit cells. Here the authors show that physical compaction of less fit cells surrounded by healthy neighbours leads to increased expression of tumour suppressor p53 in the compacted cells, causing cell death.

## Graphical abstract

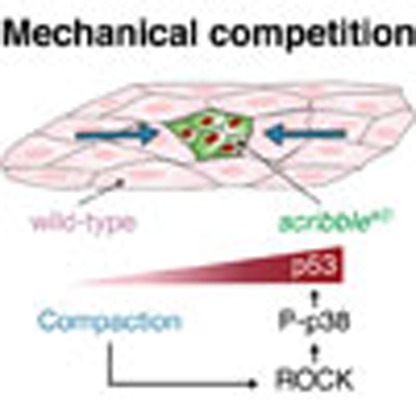

Cell competition is a remarkable phenomenon, conserved from arthropods to mammals, that causes the elimination of relatively less fit cells from tissues, helping to maintain overall tissue health^1^,^2^,^3^,^4^,^5^,^6^,^7^,^8^,^9^,^10^. Despite important advances^11^,^12^,^13^,^14^,^15^,^16^, the mechanisms that lead to the elimination of unfit cells are still little understood and it is unclear whether one or multiple pathways lead to cell killing^17^,^18^,^19^,^20^,^21^,^22^.

It has recently been reported that Madin–Darby canine kidney (MDCK) epithelial cells silenced for the polarity gene *scribble* (*scrib*^*KD*^ cells) are eliminated in the presence of wild-type MDCK cells^23^, while they are viable on their own^23^. However, the mechanisms by which *scrib*^*KD*^ cells are killed by wild-type cells are largely unknown. We therefore took advantage of this recent observation to investigate the mechanisms of cell competition.

Here we show that *scrib*^*KD*^ cells are out-competed by wild-type cells through mechanical insults rather than molecular exchange. We find that *scrib*^*KD*^ cells are hypersensitive to compaction and that this is due to elevation of baseline p53 levels, which is both necessary and sufficient to induce hypersensitivity to crowding and confer a mechanical loser status. We further show that on contact with wild-type cells, *scrib*^*KD*^ cells become compacted into a high-density arrangement and that compaction is not only required but also sufficient to eliminate *scrib*^*KD*^ cells. We also delineate the mechano-transduction cascade that leads to *scrib*^*KD*^ cell death. Specifically, we show that *scrib*^*KD*^ cells' compaction causes activation of the Rho-associated kinase (ROCK), which in turn activates p38 leading to further p53 elevation and cell death. Overall, this work demonstrates that mechanical forces can be responsible for the elimination of cells during cell competition and that p53 levels play a key role both in instructing the mechanical loser status and in the execution of mechanical cell competition.

## Results

### Compaction of *scrib* ^*KD*^ cells induces mechanical competition

**Figure 1:**
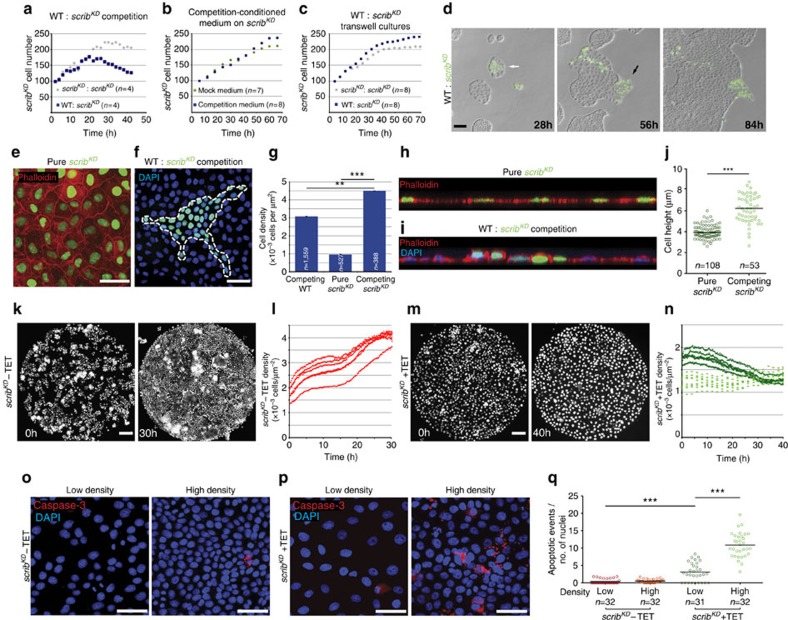
Compaction of *scrib*^*KD*^ cells is both required and sufficient for their elimination. (**a**–**c**) Quantification showing growth rate of *scrib*^*KD*^ cells from time-lapse movies of: competition versus pure cultures (**a**), pure cultures in mock conditioned versus competition conditioned medium; two biological replicates (**b**) or transwell experiments, where *scrib*^*KD*^ cells were co-cultured across transwells with control or with competing cultures; three biological replicates (**c**). Each dot represents the average of *n* fields of cells. See also Supplementary Fig. 1c. (**d**) Time course of cell competition assay between unlabelled wild-type (WT) and GFP-labelled *scrib*^*KD*^ MDCK cells. Competition is observed in surrounded *scrib*^*KD*^ cells (white arrow), but not in cells that are only contacted (black arrow), see corresponding Supplementary Movie 2. (**e**) Confluent GFP-labelled *scrib*^*KD*^cells stained with phalloidin. (**f**) Competing unlabelled WT and GFP-labelled *scrib*^*KD*^ cells counterstained with DAPI. (**g**) Quantification showing average (±s.e.m.) cell density values of confluent pure *scrib*^*KD*^ cells and subconfluent competing WT and *scrib*^*KD*^cells as in **e** and **f**. (**h**,**i**) Confocal *xz* sections of representative GFP-labelled *scrib*^*KD*^cells pure (**h**) or co-cultured with WT cells (**i**), stained with phalloidin (**h**,**i**) and DAPI (**i**). (**j**) Quantifications of cell height from images as in **h** and **i**. Black bars=median. (**k**,**m**) Representative stills from time lapse of GFP-labelled *scrib*^*KD*^ cells +/−TET growing on micropatterns (800 μm Ø), see corresponding Supplementary Movie 3. (**l**,**n**) Quantifications of cell density over time from movies as in **k** and **m**. Each dotted line corresponds to a different movie. (**o**,**p**) Cleaved Caspase-3 staining in WT (**o**) and *scrib*^*KD*^ (**p**) cells +/− compression (high density and low density, respectively). (**q**) Quantification of cell death events (cleaved Caspase-3) from images as in **o** and **p**. Data are pooled from three biological replicates. Black bars=mean; three biological replicates across two independent experiments. *n*=number of fields imaged in a single repeat (**a**–**c**,**q**) or *n*=number of cells (**g**,**j**). Scale bars, 100 μm (movie sequences) and 50 μm (immunofluorescence images) here and throughout all figures. **P<0.005, ***P<0.0005 by KS test.

It has previously been shown that *scrib*^*KD*^ MDCK cells are eliminated when co-cultured with wild-type MDCK cells through cell death and delamination (see ref. 23 and Supplementary Fig. 1a and Supplementary Movie 1, left), while monocultures of *scrib*^*KD*^ cells are viable (see ref. 23 and Supplementary Fig. 1b and Supplementary Movie 1, right). To investigate the mechanisms of *scrib*^*KD*^-mediated cell competition, we first asked whether it is mediated by soluble factors, as in other cases of *in vitro* cell competition^6^,^24^. Growth rate (doubling time) profiles showed that *scrib*^*KD*^ cells in pure cultures divide, albeit at a reduced rate, to reach a steadily maintained number (Supplementary Fig. 1d), whereas under competing conditions, their numbers collapse following initial growth (Fig. 1a). Interestingly, we found that the growth rate of *scrib*^*KD*^ cells is not affected by conditioned medium from competing cultures (Fig. 1b and Supplementary Fig. 1c). Similarly, in transwell systems that allow exchange of solutes but prevent cell contact, *scrib*^*KD*^ cells grown together with co-cultures of competing (wild-type/*scrib*^*KD*^) cells grew comparably to *scrib*^*KD*^ cells grown with other *scrib*^*KD*^ cells (Fig. 1c and Supplementary Fig. 1c). This indicated that soluble factors are not sufficient to induce cell competition and that cell contact is required. We hypothesized that cell contact enables molecular interactions essential for cell competition, as observed by others^11^,^12^. However, to our surprise, we found that sustained contact with wild-type cells is not sufficient for elimination of *scrib*^*KD*^ cells (Fig. 1d, black arrow and Supplementary Movie 2) and that *scrib*^*KD*^ clones are efficiently eliminated only when fully surrounded by wild-type cells (Fig. 1d, white arrow and Supplementary Movie 2). This suggested that a type of exchange other than molecular signalling (which would be enabled by contact) may be needed, and prompted us to look for differences between *scrib*^*KD*^ clones that were surrounded and peripheral clones that were simply contacted.

One striking feature of surrounded clones, which is not shared by peripheral clones, is that they reach a dramatically higher cell density than confluent *scrib*^*KD*^ pure cultures (Fig. 1e–g and Supplementary Fig. 1e). *scrib*^*KD*^ cells acquire a flattened morphology upon gene silencing^23^,^25^, which at confluence results in a much lower (∼1/3) final density compared with wild-type cells (Fig. 1e–g). However, *scrib*^*KD*^ clones surrounded by wild-type cells do not flatten and reach a density that is ∼4.5-fold higher than that of pure *scrib*^*KD*^ cultures (Fig. 1e–g). Furthermore, competing *scrib*^*KD*^ cells are taller than when grown in single cultures (Fig. 1h–j). Together, this indicates that as a result of their interaction with wild-type cells, *scrib*^*KD*^ cells become more compacted than in their default state and this correlates with and precedes their elimination.

We next asked whether cell compaction plays a role in *scrib*^*KD*^ cell elimination, by assessing its effect on *scrib*^*KD*^ cells in complete absence of wild-type cells. To that end, we used micropatterns to form *scrib*^*KD*^ microcultures of a defined, homogenous density and size (Ø=800 μm, ref. 26). When plated at high density, control cells that had not undergone *scribble* silencing (without tetracycline, −TET) continued to grow until they reached a maximal density of 61±4 cells per 10,000 μm^2^, which they maintained homeostatically (Fig. 1k,l and Supplementary Movie 3, left), as previously shown^27^,^28^. Remarkably, *scrib*^*KD*^ cultures (+TET) instead saw their numbers and densities drop (Fig. 1m,n, dark green and Supplementary Movie 3, right), due to a combination of increased cell death and extrusion, the same two events that lead to the elimination of *scrib*^*KD*^ cells during cell competition (Supplementary Movie 1, left and ref. 23). Notably, *scrib*^*KD*^ cells seeded at a lower density maintained that initial density (Fig. 1n, light green) indicating that the drop in cell number observed at higher density is not a general response of *scrib*^*KD*^ cells to plating. This suggested that *scrib*^*KD*^ cells are hypersensitive to compaction. To test this directly, we seeded confluent monolayers of control (−TET) or *scrib*^*KD*^ (+TET) cells on stretched polydimethylsiloxane (PDMS) substrates, which we then released to induce cell compression^27^. Control cultures showed no increase in apoptosis upon compression (Fig. 1o–q). The *scrib*^*KD*^ cells, however, displayed a 3-fold increase in apoptosis over their uncompressed baseline cell death, which was already higher than control (Fig. 1p,q). Altogether, these experiments show that *scrib*^*KD*^ cells display hypersensitivity to cell density and cannot sustain levels of crowding normally reached by wild-type cells. They further indicate that compaction of *scrib*^*KD*^ cells into higher cell densities, like those imposed on them by wild-type cells during competition, is sufficient to induce cell death. This suggests that wild-type cells eliminate *scrib*^*KD*^ cells through crowding-induced compaction. We name this new mode of selective elimination of one cell population by another due to differential sensitivity to crowding ‘mechanical cell competition', to contrast it with forms of cell competition that rely on molecular exchange.

### Corralling promotes but is not needed for *scrib* ^*KD*^ elimination

**Figure 2:**
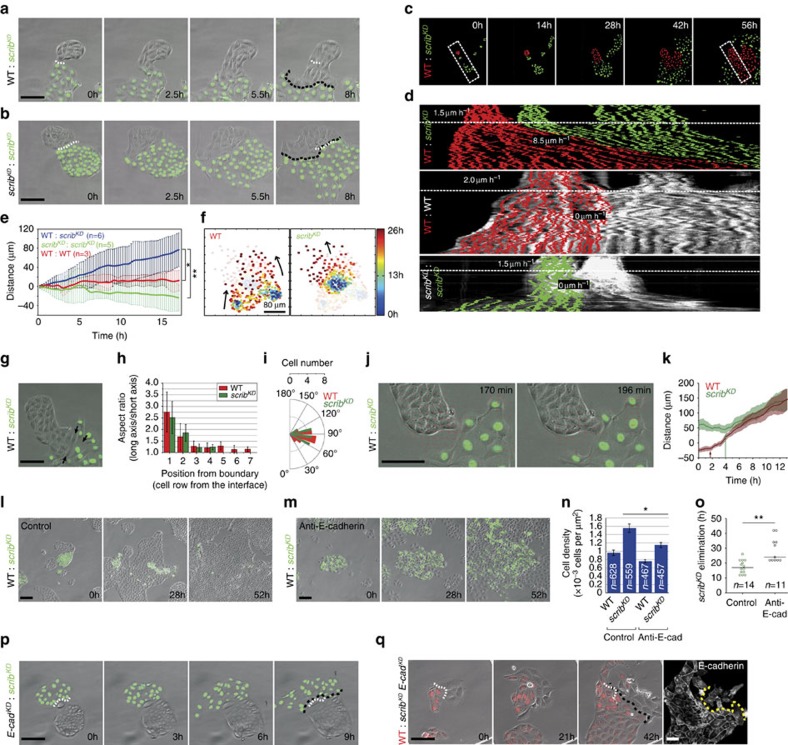
Contact-induced migration promotes compaction and cell competition. (**a**,**b**) Stills from movies of wild-type (WT) and GFP-labelled *scrib*^*KD*^ co-cultures (**a**) or *scrib*^*KD*^ homotypic cultures (**b**), see Supplementary Movie 4,5. (**c**,**d**) Kymographs (**d**) from movies as in **c**. Velocities are shown before (above dashed white line) and after contact (below line). (**e**) Plot showing displacement of the line of contact between clones from movies as in **c**. The continuous line is the position of the front average±s.d.; *n*=number of contact lines averaged. (**f**) Single-cell tracking of trajectories of WT and *scrib*^*KD*^ cells during competition. Heat-map representation shows time-resolved position of single cells. (**g**) Micrograph exemplifying cell shape change (arrows) after contact between WT and *scrib*^*KD*^ cells. (**h**) Bar plot representing aspect ratio of WT and *scrib*^*KD*^ cells as a function of distance from their contact point. *n*=50 cells of each type from three movies; error bars=s.d. (**i**) Distribution of angles between a cell's long axis and its direction of motion; *n* (WT)=18 cells; *n* (*scrib*^*KD*^)=17 cells. (**j**,**k**) PIV analysis of images at time of contact (see Supplementary Movie 6) (**j**); and quantification of cell displacements (**k**) shows WT cells begin migrating (arrows) before *scrib*^*KD*^ cells; *n*=10 cells for each type from three independent movies. Coloured lines=mean; shaded areas=s.d. (**l**–**o**) Disrupting cell junctions by E-cadherin blocking antibody and calcium removal prevents contact-induced migration (**m**), compaction (**n**) and delays competition (**o**) compared with control (**l**), see Supplementary Movie 8; error bars=s.e.m. (**p**) E-cadherin knockdown in WT cells (*E-cad*^*KD*^) prevents contact-induced migration. (**q**) E-cadherin knockdown in *scrib*^*KD*^ cells (*scrib*^*KD*^ *E-cad*^*KD*^) prevents contact-induced migration, see Supplementary Movie 9. Right panel displays anti-E-cadherin immunofluorescence at end of movie (see Supplementary Fig. 2h,i). Five independent repeats; *n*=10 events showing absence of directional migration, five were validated for E-cadherin levels and all five had WT levels. White dashed line=initial contact point; black dashed line=final contact point; yellow dashed line separates WT from *scrib*^*KD*^ *E-cad*^*KD*^ cells. *P<0.05, **P<0.005 by KS test.

As shown in Fig. 1f,g, in subconfluent competing cultures, the density of *scrib*^*KD*^ cells surpasses that of surrounding wild-type cells. This indicates that their acquired density does not simply reflect the density of the mixed culture, but is the result of an active process. To understand how *scrib*^*KD*^ cells become compacted during competition, we plated cells at low density and observed what happens when wild-type and *scrib*^*KD*^ clones first come into contact. Strikingly, we saw that, on contact, both *scrib*^*KD*^ and wild-type cells engage in collective cell migration (Fig. 2a and Supplementary Movie 4). This behaviour was specific to wild-type/*scrib*^*KD*^ encounters, as it was not observed upon homotypic encounters of either cell population (Fig. 2b and Supplementary Movie 5). Interestingly, this migration was highly directional with *scrib*^*KD*^ cells always at the migrating front and, conversely, wild-type cells always at the back. We next characterized this migratory behaviour and assessed its contribution to cell compaction and elimination. First, we repeated the above experiment using a fluorescent nuclear label in each population to facilitate cell tracking (Fig. 2c). Kymograph analysis showed rapid activation of collective cell migration at the time of contact between wild-type and *scrib*^*KD*^ cells, with both populations moving synchronously (Fig. 2d, top and Fig. 2e). By contrast, similar kymographs of homotypic collisions did not show significant cell displacement (Fig. 2d, centre and bottom; and Fig. 2e). To further characterize the features of this collective cell migration, we carried out single-cell tracking of both cell populations before and after contact. The analysis of individual cell trajectories shows that single-cell movement is faster and more persistent for both wild-type and *scrib*^*KD*^ cells upon heterotypic collision (Fig. 2f and Supplementary Fig. 2a–d). We then looked more closely at the dynamic interplay between wild-type and *scrib*^*KD*^ cells at the onset of migration. Interestingly, analysis of cell shape at the interface between the two populations revealed that both wild-type and *scrib*^*KD*^ cells become elongated at the site of contact (Fig. 2g,h), indicating that they are under anisotropic stress. Moreover, the cells in both populations move in the direction of their short axis (Fig. 2i), suggesting that they could be locally compressed. This could be the result of wild-type cells beginning migration and locally ‘piling up' against the *scrib*^*KD*^ cells. In agreement with this, particle image velocimetry (PIV) and single-cell tracking revealed that wild-type cells begin migrating towards *scrib*^*KD*^ cells on average ∼2 h before the *scrib*^*KD*^ cells start migrating away (Fig. 2j,k and Supplementary Movie 6). Together, the chronology of these events and the local cellular deformations suggest that cells might engage in a behaviour similar to the ‘chase and run' migration reported for other cell types^29^. These experiments do not distinguish whether *scrib*^*KD*^ cells are pushed by wild-type cells or, conversely, whether *scrib*^*KD*^ cells self-compact to avoid closer interaction wild-type cells. However, it is clear that as migration progresses, *scrib*^*KD*^ cells become corralled by wild-type cells and are compacted and eliminated (Supplementary Movie 7), indicating that this behaviour may facilitate compaction and outcompetition of *scrib*^*KD*^ cells.

To directly test the relevance of directional cell migration in the elimination of *scrib*^*KD*^ cells, we next sought to disrupt this behaviour. Adhesion molecules play an important role in collective cell migration^30^ and have also been implicated in ‘chase and run' among mesenchymal cells^29^. In addition, *scribble* downregulation has previously been shown to induce intracellular accumulation of E-cadherin^23^,^31^ and we found that it causes an increase of both total and cell surface E-cadherin levels (Supplementary Fig. 2e–g). We therefore reasoned that targeting E-cadherin-mediated adhesion might disrupt directional migration between the wild-type and *scrib*^*KD*^ cells. We undertook two separate approaches and found that either blocking E-cadherin function in both populations, through a combination of low calcium and addition of a blocking antibody (Fig. 2l–o and Supplementary Movie 8), or silencing E-cadherin only in wild-type cells (*E-cad*^*KD*^ (ref. 32); Fig. 2p) was sufficient to inhibit directional migration. Interestingly, disruption of E-cadherin inhibited active cell compaction (Fig. 2l,m, Supplementary Movie 8 and quantification in Fig. 2n) and resulted in delayed elimination of *scrib*^*KD*^ cells (Fig. 2l,m, Supplementary Movie 8 and quantification in Fig. 2o). However, it did not rescue the *scrib*^*KD*^ cells from cell competition, since they were eventually eliminated as the culture became progressively more crowded due to proliferation (Fig. 2l,m and Supplementary Movie 8).

Having established that E-cadherin-mediated adhesion is involved in contact-induced migration between wild-type and *scrib*^*KD*^ cells, we next asked whether the upregulation of E-cadherin observed in *scrib*^*KD*^ cells (Supplementary Fig. 2e–g) plays a role in this directional cell movement. We therefore generated an inducible double *scrib*^*KD*^ *E-cadherin* knockdown cell line (*scrib*^*KD*^ *E-cad*^*KD*^) and specifically selected clones that displayed partial silencing, enough to bring E-cadherin down to wild-type levels (Fig. 2q, right panel and Supplementary Fig. 2h,i). Indeed, partial downregulation of E-cadherin inhibited contact-induced migration, suggesting that high E-cadherin levels in the *scrib*^*KD*^ cells are required for this process (Fig. 2q and Supplementary Movie 9). As expected, these clones were still outcompeted by wild-type cells (Supplementary Fig. 2j). In contrast, we found that E-cadherin upregulation alone is not sufficient to cause contact-induced migration, as cells overexpressing E-cadherin at levels comparable to those of *scrib*^*KD*^ cells (Supplementary Fig. 2k) did not engage in directional cell migration with wild-type cells upon contact (Supplementary Fig. 2l). Altogether, we conclude that directional cell migration, by enabling corralling and active compaction of *scrib*^*KD*^ cells, promotes but is not required for mechanical cell competition, and that E-cadherin is necessary for corralling and active compaction but it does not impact on loser cell status.

### p53 is activated in *scrib* ^*KD*^ cells before cell competition

**Figure 3:**
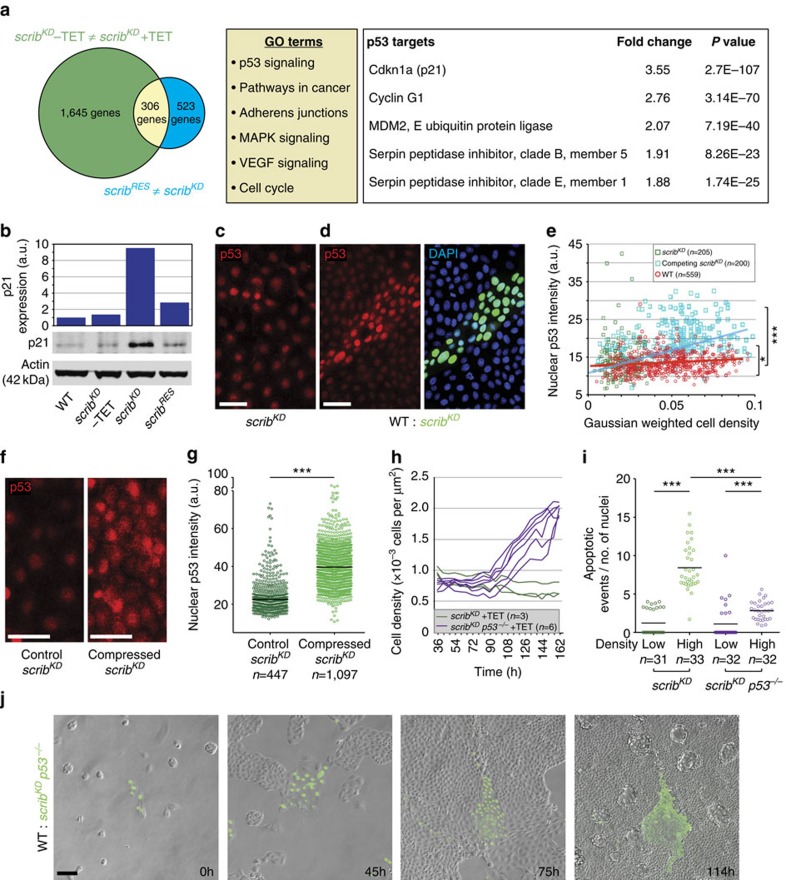
p53 is activated in *scrib*^*KD*^ cells before competition and further p53 elevation upon compaction causes competition-induced cell death. (**a**) Left, transcriptional profiling of *scrib*^*KD*^ cells without tetracycline (TET) versus *scrib*^*KD*^ cells with TET (green) and of *scrib*^*KD*^ cells with TET versus *scrib*^*KD*^ cells resistant to competition (*scrib*^*RES*^) with TET (blue), and corresponding intersection of differentially expressed genes (yellow). Middle, list of pathways functionally enriched in the yellow intersection. Right, list of p53 targets found in this intersection and corresponding fold change difference between *scrib*^*KD*^ cells −/+TET. Three biological replicates for *scrib*^*KD*^ cells −/+TET and two biological replicates for *scrib*^*RES*^ cells +TET. (**b**) Western blot against p21 with LICOR quantifications and normalization to Actin. (**c**,**d**) p53 staining of pure *scrib*^*KD*^ cells (**c**) and of co-cultures of GFP-labelled *scrib*^*KD*^ and wild-type (WT) cells (**d**). (**e**) Graph showing single-cell nuclear p53 intensity plotted against cell density from images as in **c** and **d**; *P<0.05 by KS test comparing p53 levels in pure *scrib*^*KD*^ versus WT; ***P<0.0005 by KS test comparing competing *scrib*^*KD*^ versus WT. Non-parametric Spearman correlation; red line=WT cells; blue line=competing *scrib*^*KD*^ cells. (**f**,**g**) p53 staining in pure *scrib*^*KD*^ cells on PDMS substrate +/− compression (**f**) and quantification showing single-cell nuclear p53 intensity (**g**) from images as in **f**; black bars=median. (**h**) Time-resolved density measurement of growing *scrib*^*KD*^ cells and *scrib*^*KD*^ *p53*^−/−^ cells. (**i**) Quantification of cell death (cleaved Caspase-3) for *scrib*^*KD*^ cells versus *scrib*^*KD*^ *p53*^−/−^ cells on PDMS substrate +/− compression; black bars=mean; data from three biological replicates across two independent experiments. (**j**) Stills from time-lapse movies of WT and GFP-labelled *scrib*^*KD*^ *p53*^−/−^ co-cultures, see corresponding Supplementary Movie 11; *n*=cell number in **e** and **g** or *n*=number of imaged fields of cells in **h** and **i**. a.u.=arbitrary units, here and throughout all the figures. *P<0.05, ***P<0.0005 by KS test.

Key to the outcompetition of *scrib*^*KD*^ cells is their hypersensitivity to compaction (Fig. 1k–q). To identify genes and pathways involved in this behaviour, we carried out transcriptional profiling of *scrib*^*KD*^ cells (*scrib*^*KD*^ +TET) and compared it with the transcriptomes of control MDCK cells (*scrib*^*KD*^ −TET) and, importantly, of an isolate of *scrib*^*KD*^ cells that is resistant to cell competition (*scrib*^*RES*^). Despite maintaining *scribble* gene knockdown (Supplementary Fig. 3a), the *scrib*^*RES*^ cells do not display elevated E-cadherin (Supplementary Fig. 3b), do not engage in contact-induced migration with wild-type cells (Supplementary Fig. 3c) and are not outcompeted in cell competition assays (Supplementary Fig. 3d and Supplementary Movie 10). Notwithstanding these fundamental differences, we observed that the *scrib*^*KD*^ transcriptome is still substantially closer to *scrib*^*RES*^ (with 523 differentially expressed genes) than to control (*scrib*^*KD*^ −TET) cells (with 1,645 differentially expressed genes; Fig. 3a, left and Supplementary Data 1 and 2). This allowed us to rule out all genes that are differentially expressed in *scrib*^*KD*^ versus wild-type cells but are similarly expressed between *scrib*^*KD*^ and *scrib*^*RES*^, as these are clearly not sufficient to induce cell competition. Instead, we focused on the small intersection of 306 genes that are differentially expressed between wild-type and *scrib*^*KD*^ cells but are also different between *scrib*^*KD*^ and *scrib*^*RES*^ cells (Fig. 3a and Supplementary Data 3).

Gene Ontology term enrichment analysis highlighted p53 signalling as the top functionally enriched pathway (Fig. 3a, middle). A number of known p53 target genes were moderately upregulated in *scrib*^*KD*^ cells, suggesting p53 activation (Fig. 3a, right). Consistent with this, we found that p21 (a known p53 target^33^ and the most highly upregulated p53 target in our gene list; Fig. 3a) is specifically upregulated upon *scribble* knockdown in *scrib*^*KD*^ cells (Fig. 3b, Supplementary Fig. 3e and Supplementary Fig. 4d,e) but not in *scrib*^*RES*^ cells (Fig. 3b) and that p53 levels are higher in *scrib*^*KD*^ cells than in wild-type cells (Fig. 3c,d and Supplementary Fig. 3f), confirming pathway activation. Thus, *scrib*^*KD*^ cells have high baseline p53 activity and this correlates with their loser status.

### p53 is further elevated in *scrib* ^*KD*^ cells by compaction

Next we asked whether cell competition affects p53 activity. Interestingly, we found that in competing conditions, p53 levels in *scrib*^*KD*^ cells increase above their already elevated baseline level, in a way that correlates with the degree of cell compaction (Fig. 3c–e; *r*=0.56 by non-parametric Spearman correlation). This suggested that compaction increases p53 levels or, conversely, that higher p53 levels enable compaction. To distinguish between these two possibilities, we looked at how compression alone affects p53, using deformable PDMS substrates as before (Fig. 1o–q). Importantly, we found that compression alone causes an increase in p53 levels (Fig. 3f,g), as seen during competition. This indicates that cell competition induces further p53 activation in the *scrib*^*KD*^ cells via compaction-induced mechanical stress.

Given that p53 is upregulated during cell competition, we next sought to ask whether it contributes to this process. Therefore we mutated p53 in *scrib*^*KD*^ cells by CRISPR-mediated mutagenesis (*scrib*^*KD*^ *p53*^−/−^ cells, Supplementary Fig. 4a,b). The *scrib*^*KD*^ *p53*^−/−^ cells failed to upregulate p21 following ultraviolet irradiation (Supplementary Fig. 4b) or *scribble* knockdown (Supplementary Fig. 4c–e), confirming functional p53 inactivation. Strikingly, we found that p53 inactivation in *scrib*^*KD*^ *p53*^−/−^ cells was sufficient to partially rescue their low homeostatic cell density (Fig. 3h) and their hypersensitivity to compaction (Fig. 3i). Remarkably, genetic (using *scrib*^*KD*^ *p53*^−/−^ cells) or chemical (using the inhibitor Pifithrin-α) inhibition of p53 in *scrib*^*KD*^ cells was also sufficient to prevent their outcompetition (Fig. 3j and Supplementary Movie 11; Supplementary Fig. 4f). Furthermore, *scrib*^*KD*^ *p53*^−/−^ cells maintained high E-cadherin levels (Supplementary Fig. 4g) and still displayed contact-induced migration (Supplementary Fig. 4h), demonstrating again that corralling is not sufficient for competition. Altogether, we conclude that high baseline p53 activity in *scrib*^*KD*^ cells is associated with their loser status and is required for them to acquire a low homeostatic cell density and hypersensitivity to compaction, two key features of the mechanical loser status.

Interestingly, though it has long been established that *scribble*^−/−^ cells are eliminated by cell competition in *Drosophila*^34^,^35^, when we tested whether this might happen via mechanical insults in wing imaginal discs we found that, unlike in MDCK cells, *scribble*^−/−^ wing disc cells did not upregulate E-cadherin (Supplementary Fig. 5a,b) and their outcompetition was not rescued by p53 inhibition (Supplementary Fig. 5c). It is possible that *scribble*^−/−^ cells are not eliminated by mechanical cell competition in *Drosophila* or that the function of Scribble or p53 may not be conserved in this process. Alternatively and perhaps more likely, mechanical cell competition may be redundant with other mechanisms of cell competition that have been described to target *scribble*^−/−^ cells in that system^36^,^37^.

### ROCK activates p38 leading to p53 elevation and cell death

**Figure 4:**
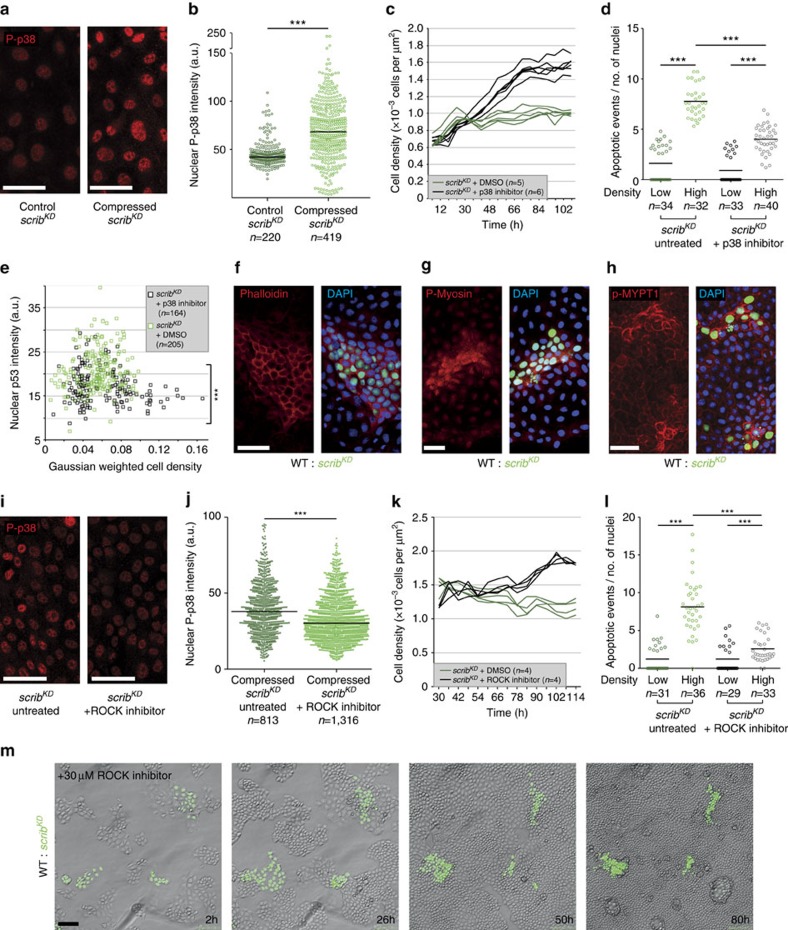
During mechanical cell competition ROCK activates p38 leading to p53 elevation. (**a**) Phosphorylated p38 (P-p38) staining in pure *scrib*^*KD*^ cells +/− compression. (**b**) Single-cell nuclear P-p38 intensity from images as in **a**; black bars=median. (**c**) Time-resolved density measurement of growing *scrib*^*KD*^ cells +/− p38 inhibitor. (**d**) Quantification of cell death (cleaved Caspase-3) of *scrib*^*KD*^ cells with or without compression and +/− p38 inhibitor; black bars=mean; pooled data from three biological replicates across two independent experiments. (**e**) Single-cell nuclear p53 signal intensity in competing *scrib*^*KD*^ cells +/− p38 inhibitor, plotted against cell density. (**f**–**h**) F-Actin (phalloidin-stained, **f**), phosphorylated Myosin-II (P-Myosin, **g**) and phosphorylated MYPT1 (p-MYPT1, **h**) are elevated in compacted GFP-labelled *scrib*^*KD*^ cells compared with wild-type (WT) cells during competition. (**i**) P-p38 staining in compressed *scrib*^*KD*^ cells +/− ROCK inhibitor. (**j**) Single-cell nuclear P-p38 intensity from images as in **i**; black bars=median. (**k**) Time-resolved density measurement of growing *scrib*^*KD*^ cells +/− ROCK inhibitor; two independent repeats. (**l**) Quantification of cell death (cleaved Caspase-3) in *scrib*^*KD*^ cells with or without compression and +/− ROCK inhibitor; black bars=mean; pooled data from three biological replicates across two independent experiments. (**m**) Stills from time-lapse movies of WT and GFP-labelled *scrib*^*KD*^ co-cultures treated with ROCK inhibitor (30 μM), see corresponding Supplementary Movie 12; *n*=cell number in **b**,**e** and **j** or *n*=number of imaged fields of cells in **c**,**d**,**k** and **l**. ***P<0.0005 by KS test.

We next wondered how mechanical stress might lead to p53 activation. A potential candidate was p38 signalling, as it is required for *scrib*^*KD*^ cell competition^23^, is known to promote p53 activity^38^,^39^,^40^ and has also been involved in the response to mechanical stress^41^. Consistent with an involvement of p38, we found that compression alone causes an increase in active phosphorylated (T180/Y182) p38 (ref. 38; P-p38) in *scrib*^*KD*^ cells (Fig. 4a,b) and that chemical inhibition of this pathway partially rescues both the homeostatic density of *scrib*^*KD*^ cells (Fig. 4c) and their compaction hypersensitivity (Fig. 4d). Moreover, the upregulation of p53 in competing *scrib*^*KD*^cells was reduced by p38 inhibition (Fig. 4e). We conclude that in *scrib*^*KD*^ cells, compression induces p53 via activation of p38.

We next asked how compression of *scrib*^*KD*^ cells causes p38 activation. We monitored cytoskeletal changes induced by compression and found that both cortical Actin (by phalloidin staining) and active phosphorylated-myosin (P-Myosin) are upregulated in compacted *scrib*^*KD*^ cells during competition (Fig. 4f,g). Since the cytoskeletal regulator ROCK^42^ is one of the main kinases responsible for Myosin phosphorylation, this suggested that ROCK might be activated. Indeed P-Myosin upregulation was reduced in the presence of a ROCK inhibitor (Supplementary Fig. 6a,b) and the ROCK target phospho-MYPT1 (ref. 43; p-MYPT1) was also elevated in compacted *scrib*^*KD*^ cells (Fig. 4h), indicating that ROCK is activated in compacted *scrib*^*KD*^ cells. This was potentially relevant because ROCK has been shown to phosphorylate p38 (ref. 44). Thus, to ask whether ROCK is upstream of p38 activation, we compressed pure cultures of *scrib*^*KD*^ cells in the presence or absence of a ROCK inhibitor and looked at P-p38 levels. ROCK inhibition led to a partial reduction of P-p38 levels, thus placing ROCK upstream of p38 signalling (Fig. 4i,j). In addition, ROCK inhibition was sufficient to partially rescue the homeostatic density of *scrib*^*KD*^ cells (Fig. 4k) and compression-induced cell death (Fig. 4l). Importantly, inhibition of ROCK was also sufficient to prevent the out-competition of *scrib*^*KD*^ cells, with no appreciable cell death observed even though cells were compacted far beyond standard competition densities (Fig. 4m and Supplementary Movie 12). Altogether, these experiments indicate that mechanical cell competition is caused by compaction-induced ROCK activation, which activates p38, leading to p53 elevation and cell death. Interestingly, ROCK has also been implicated in apical extrusion of dying or crowded MDCK cells^45^,^46^. However, in the case of Scribble competition, ROCK has a different function, as it is involved in p53 activation and cell death. In addition, while in that context ROCK was activated by S1P2 and Piezo signalling^45^,^46^, inhibition of these pathways had no effect on Scribble cell competition (Supplementary Fig. 7a,b), suggesting a distinct upstream activation mechanism.

### p53 activation turns wild-type cells into mechanical losers

**Figure 5:**
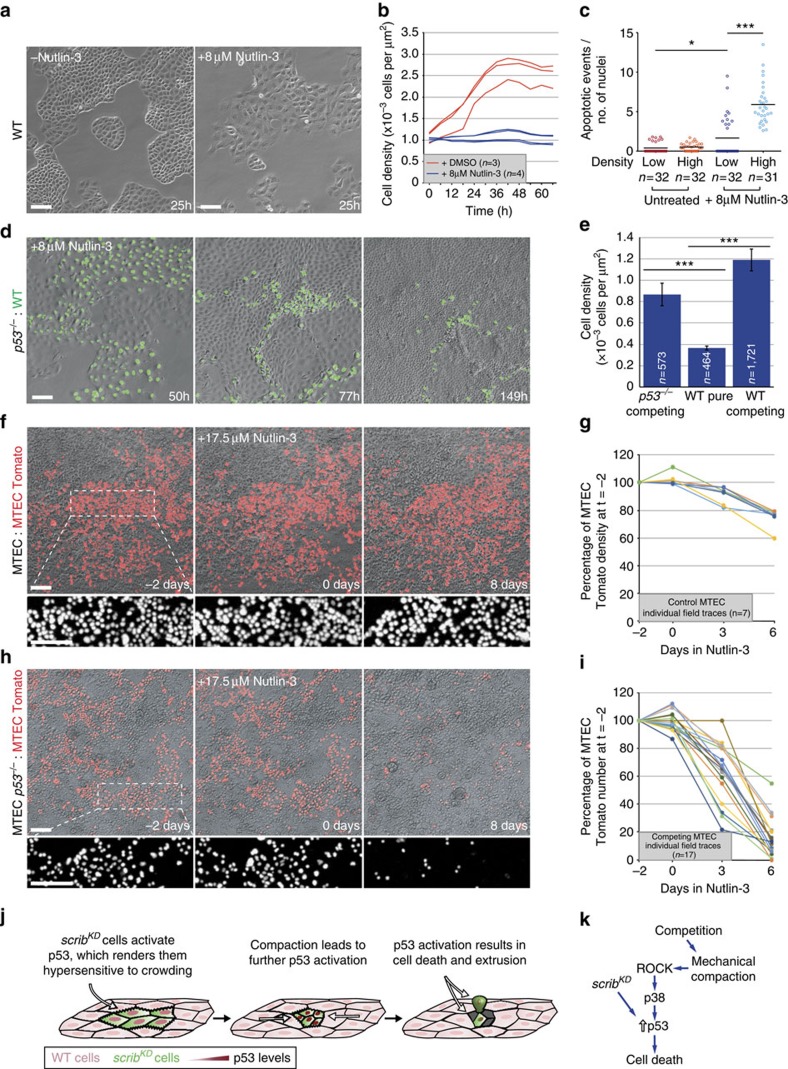
p53 activation is sufficient to induce crowding hypersensitivity and mechanical cell competition. (**a**) Addition of Nutlin-3 (8 μM) causes flattening of wild-type (WT) MDCK cells; *n*=4 fields per repeat. (**b**) Time-resolved cell density measurement of growing WT MDCK cells +/− Nutlin-3 (8 μM). (**c**) Quantification of cell death (cleaved Caspase-3) of WT MDCK cells with and without compression and +/− Nutlin-3 (8 μM); black bars=mean; three biological replicates from two independent experiments. (**d**) Stills from time-lapse movies of WT and *p53*^−/−^ MDCK co-cultures with Nutlin-3 (8 μM) see corresponding Supplementary Movie 13. (**e**) Cell density measurement from movies as in **d**; mean±s.e.m. (**f**) Stills from time-lapse movies of primary cultures of unlabelled and Tomato-labelled WT MTECs (see corresponding Supplementary Movie 14). Nutlin-3 (17.5 μM) was added at *t*=0. (**g**) Time-resolved cell density measurement from movies as in **f** of WT MTECs before and after Nutlin-3 (17.5 μM) addition. (**h**) Stills from time-lapse movies of primary cultures of unlabelled *p53*^−/−^ and Tomato-labelled WT MTECs (see corresponding Supplementary Movie 15). Nutlin-3 (17.5 μM) was added at *t*=0. (**i**) Time-resolved measurement of cell number from movies as in **h** of WT MTECs before and after Nutlin-3 (17.5 μM) addition. (**j**,**k**) Model of mechanical cell competition of *scrib*^*KD*^ cells; *n*=cell number in **e** or *n*=number of imaged fields of cells in **b**,**c**,**g** and **i**. For **c** and **e**, data are pooled from three biological replicates. *P<0.05, ***P<0.0005 by KS test.

Finally, we decided to address how *scribble* knockdown earmarks cells as losers. Our data indicated that a loser cell status is not an obligate outcome of *scribble* silencing, as we could isolate *scrib*^*KD*^ cells that are competition resistant (*scrib*^*RES*^ cells; Supplementary Fig. 3d). Instead, our data showed that p53 is necessary for competition and that mild p53 elevation is required for *scrib*^*KD*^ cells to acquire key features of the mechanical loser status (Fig. 3h,i). This prompted us to investigate whether p53 might actually be sufficient to induce the mechanical loser status and cell competition. Nutlin-3, a chemical inhibitor of the E3 ubiquitin ligase MDM2 (Mouse Double Minute 2 (ref. 47)), activates p53 in a dose-dependent manner. This allowed us to establish conditions to induce mild p53 activation in wild-type MDCK cells. Strikingly, low-level p53 activation was sufficient to induce cell flattening (Fig. 5a) and to lower the homeostatic density of wild-type cells (Fig. 5b). Remarkably, Nutlin-3 treatment was also sufficient to induce compaction hypersensitivity (Fig. 5c). Thus, mild p53 activation is sufficient to induce in wild-type cells all the features that we observed in *scrib*^*KD*^ cells that are hallmarks of their mechanical loser status. Therefore, we next asked whether differential p53 levels are sufficient to induce mechanical cell competition. To test this, we generated a p53 knockout cell line (*p53*^−/−^), which we then co-cultured with wild-type cells. Not surprisingly, simply mixing wild-type and *p53*^−/−^ cells was not sufficient to induce competition (Supplementary Fig. 8a). Strikingly however, mild p53 activation in wild-type cells, by Nutlin-3 addition at sublethal doses, caused the elimination of these cells specifically in the co-culture, but not when they were cultured alone (Fig. 5d, Supplementary Fig. 8b and Supplementary Movie 13). Importantly, p53-induced cell competition was indistinguishable from *scribble* cell competition, as it resulted in loser cell compaction (Fig. 5e) and only caused elimination when cells were compacted by *p53*^−/−^ cells (Fig. 5d). Overall, these results demonstrate that mild elevation of p53 is sufficient to phenocopy Scribble cell competition and induce both hypersensitivity to cell crowding and the mechanical loser status in otherwise wild-type cells.

To demonstrate the existence of p53-mediated mechanical cell competition beyond the MDCK experimental paradigm, we turned to primary cultures of epithelial cells, specifically to mouse tracheal epithelial cells (MTECs), and looked for evidence of this process. Under normal conditions, confluent MTEC monolayers show little proliferation or change in cell density (Fig. 5f,g, compare first and second time points, and Supplementary Movie 14, left, before Nutlin-3 addition). Interestingly however, when we induced mild elevation of p53 (by Nutlin-3 treatment) in these cultures, we found that this results in a 26% average reduction in cell density, accompanied by cell extrusion, suggesting acquired hypersensitivity to crowding (Fig. 5f,g, compare before and after Nutlin-3 addition, and Supplementary Movie 14, left, after Nutlin-3 addition). To test whether differential levels of p53 could induce cell competition in MTECs, we mixed Tomato-labelled wild-type MTECs (MTEC Tomato; from *Rosa*26R-nTomato-nGFP mice^48^) either with unlabelled wild-type MTECs or with unlabelled p53 null MTECs (MTEC *p53*^−/−^; from p53 null mice). Before Nutlin-3 addition, the proportion of wild-type (Tomato) MTECs in the monolayer did not change for several days when co-cultured with either *p53*^−/−^ cells or more wild-type cells (Fig. 5f–i, first two time points, and Supplementary Movie 14, before Nutlin-3 addition). However and strikingly, Nutlin-3 addition induced robust cell competition specifically in wild-type/*p53*^−/−^ MTEC co-cultures, causing the number of wild-type cells to plummet within 6 days to ∼17% of their starting number, with pronounced cell death and fragmentation (Fig. 5h,i and Supplementary Movie 14, right). Thus, mild p53 elevation is sufficient to induce crowding hypersensitivity and mechanical cell competition in MTEC cultures.

## Discussion

In summary, our work demonstrates that in addition to molecular signals, cells use mechanical means to compete (see model in Fig. 5j,k), a concept that had previously only been speculated about and proposed on theoretical grounds^19^,^49^. We show that hypersensitivity to crowding provides an additional route to the loser cell status, leading to mechanical cell competition and the elimination of loser cells. One interesting prediction of our findings is that they suggest that competition could take place between genetically identical cells if they are endowed with differential sensitivity to mechanical stress or if they reside in tissues with varying mechanical properties^50^,^51^, a hypothesis that should be investigated. In addition, given the involvement of p53, a general sensor of cell stress, we suggest that mechanical cell competition may be widespread among the damaged cells. Moreover, future work should also examine the implications of our findings on the behaviour of cancer cells. Since *p53* is one of the most commonly mutated genes in cancer^52^, our findings suggest that its loss could enable neoplastic cells (*scribble* is a tumour-suppressor gene^53^,^54^) to evade mechanical cell competition. Identifying the physiological contexts where mechanical cell competition plays a role may help better understand tissue biology and potentially cancer formation.

*Note added in proof*: Evidence of mechanical competition in *Drosophila* was reported just prior to acceptance of this work^55^.

## Methods

### Antibodies and materials

For immunofluorescence we used:

Rabbit anti-p53 antibody (1:750, Cell Signaling Technology #9382), rabbit anti-p21 antibody (1:200, Santa Cruz sc-397). In functional tests, both antibodies show an increase in staining intensity +ultraviolet-C treatment and a reduction or complete absence of staining in *scrib*^*KD*^ *p53*^−/−^ and *p53*^−/−^ cells (Supplementary Fig. 4b). In addition, the anti-p21 antibody is documented to cross-react in canine. Mouse anti-E-cadherin antibody (1:600 total stain or 1:200 surface stain^56^), rabbit anti-E-cadherin antibody (1:500 total stain or 1:50 surface stain^57^), both documented to cross-react in canine. Rabbit anti-P-p38 MAPK (T180/Y182) antibody (1:50, Cell Signaling Technology #9215) was used as in Norman *et al*.^23^ Rabbit anti-P-Myosin light chain (phospho S20) antibody (1:100, Abcam ab2480), predicted to cross-react with all mammals. Goat p-MYPT1 (Thr 853) (1:50, Santa Cruz sc-17432) antibody, documented to cross-react in canine. Rabbit anti-cleaved Caspase-3 antibody (1:200, Cell Signaling Technology #9661s), predicted to cross-react in canine based on 100% sequence homology. DAPI (1 μg ml^−1^ Invitrogen); Alexa Fluor conjugated secondary antibodies (1:1,000, Invitrogen); Alexa Fluor-568 and Alexa Fluor-647 conjugated phalloidin (1:40, Invitrogen).

For western blotting we used:

Rabbit anti-p53 antibody (1:1000, Cell Signaling Technology #9382), rabbit anti-p21 antibody (1:500, Santa Cruz sc-397). Goat anti-Scribble antibody (1:500, Santa Cruz sc-11048), functional tests show a decrease in signal intensity in *scrib*^*KD*^ cells +TET and it is documented to cross-react in canine (Supplementary Fig. 3a). Mouse anti-E-cadherin antibody (1:1,000; (ref. 56)), rabbit anti-β-tubulin antibody (1:50,000, Abcam ab6046), HRP conjugated secondary antibodies (1:3,000, Bio-Rad) and IRDye infrared fluorescent dyes (1:10,000, LI-COR).

Inhibitors and treatments were used at the following concentrations: Pifithrin-α (Sigma) 10 μM, p38 inhibitor SB202190 (Calbiochem) 10 μM, ROCK inhibitor Y27632 (Sigma) 30 μM, Piezo inhibitor gadolinium III chloride (Sigma) 100 μM, S1P2 inhibitor JTE013 (Tocris Bioscience) 10 μM, tetracycline (Sigma) 5 μg ml^−1^ (except for pre-treating *scrib*^*KD*^ cells for RNA-seq analysis where it was used at 10 μg ml^−1^), doxycycline (Sigma) 1 μg ml^−1^, Nutlin-3 (Cayman Chemicals) was added as specified.

### Cell culture and plasmids

The cell lines used in this publication are not listed in the database of misidentified cell lines maintained by ICLAC. MDCK cell lines were authenticated in our laboratory by RNA sequencing, which confirmed their canine origin. All cell lines used in this publication have been tested in our laboratory and were found to be negative for mycoplasma infection (EZ PCR Mycoplasma Test Kit, Geneflow).

Wild-type MDCK, MDCK-pTR *E-cadherin* shRNA (*Ecad*^*KD*^)^32^ and MDCK-pTR *scribble* shRNA (*scrib*^*KD*^) cells^23^ were a kind gift from Yasuyuki Fujita. Wild-type and *E-cad*^*KD*^ MCDK cells were cultured in DMEM (21885; Invitrogen) supplemented with 10% fetal bovine serum (FBS, Invitrogen) in a humidified incubator at 37 °C with 5% CO_2_. The *scrib*^*KD*^ cells were cultured as described for wild-type cells with the addition of blasticidin 50 μg ml^−1^ (Sigma) and G418 800 μg ml^−1^ (Invitrogen) to the culture media. To establish MDCK cell lines that stably express a nuclear green fluorescent protein (GFP) or red fluorescent protein (RFP) marker, we used a modified pGIPZ-turboGFP-Puro (Thermo Scientific) plasmid where we replaced turbo-GFP with either GFP-NLS or RFP-NLS. Selection following infection was carried out with puromycin (0.65 μg ml^−1^, Sigma).

Resistant *scrib*^*KD*^ MDCK cells (*scrib*^*RES*^) were generated by culturing MDCK-pTR *scribble* shRNA cells in 5 μg ml^−1^ tetracycline for 20 days. The resulting final population was expanded in the absence of tetracycline and tested for efficient Scribble knockdown by western blotting.

The MDCK *p53*^−/−^ and *scrib*^*KD*^ *p53*^−/−^ cells were generated using Cas9 D10A CRISPR technology. sgRNAs against canine TP53 were manually designed following published guidelines^58^

(p53_CRISPR#1_Fw: 5′-GCAGAAGTGGCTGGCATCCT-3′, p53_CRISPR#2_Fw: 5′-CCCTGGACCGGCCCCCTCC-3′).

sgRNAs were individually cloned into the PX461 vector^58^ and the pair was co-transfected into recipient cells. Pools of both *p53*^−/−^ and *scrib*^*KD*^ *p53*^−/−^ cells were generated by functional selection with Nutlin-3 (20–30 μM) for 5–7 days and either used immediately or expanded from single cells. p53 knockout was verified by functional tests.

To knock down E-cadherin expression in MDCK *scribble* shRNA cells (*scrib*^*KD*^ *E-cad*^*KD*^), the recipient cells were stably transfected with a modified version (pSUPERIOR.hygro+gfp *E-cadherin* shRNA) of a plasmid provided by Yasuyuki Fujita (pSUPERIOR.neo+gfp *E-cadherin* shRNA^32^). Selection was carried out with Hygromycin B (75 μg ml^−1^, Invitrogen). To overexpress E-cadherin in wild-type MDCK cells, the E-cadherin-GFP cDNA^59^ was introduced into a modified pTRIPZ-turboRFP-Puro plasmid (Thermo Scientific) in which turboRFP was replaced by RFP-NLS (pTRIPZ-RFP-NLS-Puro). The cells infected with the pTRIPZ ECAD-GFP-P2A-RFP-NLS construct express doxycycline-inducible E-cadherin-GFP and RFP-NLS as a single transcript. Selection following infection was carried out with puromycin (0.65 μg ml^−1^, Sigma).

Primary mouse tracheal epithelial cells (MTECs) were obtained from 5-month-old animals from 26R-Tomato (*Gt(ROSA)26Sor*^tm1(CAG-tdTomato*,-EGFP*)Ees^) and p53-null (*Trp53*^*tm1tyj*^) strains, both of C57BL/6 background, using a protocol adapted from published methods^60^. Tracheas were dissected from the larynx to the bronchial main branches and collected in ice-cold DMEM:F12 (11330-32; Invitrogen) supplemented with a solution of 100 units ml^−1^ penicillin and 100 μg ml^−1^ streptomycin (Invitrogen). The muscle, vascular tissue and glands were then removed and the trachea cut into three to four rings. Each fragment was washed in phosphate-buffered saline (PBS) and then incubated in Dispase (BD Biosciences) at 7.5 Caseinolytic Units in PBS (total volume 450 μl per trachea) for 25 min at room temperature (RT). Tracheal fragments were then transferred into ice-cold DMEM:F12 and the sheets of epithelial tissue were peeled off. The epithelial sheets and medium were transferred to an ice-cold 1.5 ml tube, and pelleted twice at 500 *g* for 3 min with a PBS wash in between. The pellets were resuspended in 0.05% TE (Invitrogen) supplemented with 5 mM EDTA for 30 min at 37 °C. Five hundred microlitres of DMEM:F12 supplemented with 5% FBS was added to stop the reaction. The cells were pelleted (500 *g*, 3 min), resuspended in MTEC/Plus media and plated on collagen-coated (50 μg ml^−1^ rat tail collagen I (BD Biosciences)/0.02 M acetic acid) 24-well tissue culture inserts (BD Falcon) in MTEC/Plus media at approximately 5 × 10^4^ cells per insert. The MTEC cells were cultured in MTEC/Plus media consisting of: DMEM:F12 basal media supplemented with a solution of 100 units ml^−1^ penicillin and 100 μg ml^−1^ streptomycin, 10 μg ml^−1^ insulin (Invitrogen), 5.5 μg ml^−1^ transferrin (Invitrogen), 6.7 μg ml^−1^ selenium (Invitrogen), 0.1 μg ml^−1^ cholera toxin (Sigma), 25 ng ml^−1^ epidermal growth factor (R&D Systems), 30 μg ml^−1^ bovine pituitary extract (Invitrogen), 5% FBS, 15 mM HEPES and 0.01 μM freshly added retinoic acid (Sigma) in a humidified incubator at 37 °C with 5% CO_2_.

### Conditioned media and transwell assays

For competition-conditioned medium experiments, media were conditioned for 48 h by cultures of GFP-labelled *scrib*^*KD*^ and wild-type cells with (competition condition) or without (mock condition) tetracycline. Recipient GFP-labelled *scrib*^*KD*^ cells were pre-treated with tetracycline for 24 h and 10,000 cells per well were seeded into 24-well plates with 1 ml of conditioned medium. For transwell assays, GFP-labelled *scrib*^*KD*^ cells (10,000 per well) were seeded at the bottom of a 24-well plate. Co-cultures at a ratio of 1:10 (GFP-labelled *scrib*^*KD*^: wild-type or GFP-labelled *scrib*^*KD*^: *scrib*^*KD*^) were plated onto a transwell insert (3.0 μm pore size-polyester membrane, Corning) at 1,500 cells per insert). All *scrib*^*KD*^ and GFP-labelled *scrib*^*KD*^ cells were pre-treated for 24 h with tetracycline before plating.

### Fences system

Where applicable, immunofluorescence of cell competition was carried out in a 24-well plate using ‘fences' (Aix Scientifics, http://www.aix-scientifics.co.uk/en/fences.html). The system allows two different populations of cells to be seeded on either side of a silicone barrier, thus allowing experimental and control conditions to be cultured, treated, imaged and (where applicable) stained within the same well/coverslip.

### Cell competition and contact-induced migration assays

Cell competition assays on MDCK cells were carried out in 24-well plate fences. Control cultures were plated in the centre of the fence (1,000 cells per fence) at a ratio of 1:10 GFP-labelled *scrib*^*KD*^: *scrib*^*KD*^. Competition cultures were seeded on the outside of the barrier (8,000 cells per fence) at a ratio of 1:10, *scrib*^*KD*^: wild-type cells. The fences were removed approximately 5 h after plating and the culture medium was replaced with or without addition of tetracycline. Twenty hours later, the culture medium was replaced with phenol red-free DMEM (+10% FBS and 1% L-glutamine, Invitrogen) with or without tetracycline. Where specified, the chemical inhibitors were also added at this point, except for the ROCK, Piezo and S1P2 inhibitors, which were added after an additional 24 h. Live imaging was started 2–4 h after the final media change and was continued for at least 50 h with regular media changes every 2–3 days.

For cell migration assays, the cells were seeded as for competition assays, except that they were plated at a lower density (1,000 cells in the centre of the fence, 2,000–5,000 cells in the outer chamber) and imaged more frequently (every 10 min to every 2 h). Alternatively, where specified, cells for competition or migration assays were plated on gridded tissue culture plates (μ-Dish 35 mm Grid-500, Ibidi) at 6,250 cells per plate. This allowed us to find specific clones of cells for immunofluorescence analysis after live imaging had finished. Co-cultures, unless otherwise specified, were plated at a ratio of 1:10 (*scrib*^*KD*^: wild-type).

The MTEC cells were plated in collagen-coated tissue culture inserts and allowed to grow for approximately 2 weeks until they reached homeostatic density before commencing experiments. In control cultures, wild-type (unlabelled) cells were plated with wild-type *Rosa*26R-Tomato (nuclear red) cells (MTEC Tomato)^48^ cells at a 2:1 ratio. In competition cultures, unlabelled p53-null cells (MTEC *p53*^−/−^) and wild-type *Rosa*26R-Tomato cells (MTEC Tomato) were plated at a 2:1 ratio. Nutlin-3 was added at 17.5 μM on day 3 of live imaging. The medium was changed every 2 days.

### Homeostatic cell density assays

These assays were carried out in 96-well plates. GFP-labelled *scrib*^*KD*^ and *scrib*^*KD*^ *p53*^−/−^ cells (8,000–12,000 cells per plate) were pre-treated with tetracycline for 12 h before plating where applicable, in the presence or absence of inhibitors. Where applicable, Nutlin-3 was added 24 h after plating. The whole well was then imaged and the individual images were stitched together for further analysis to obtain data on total cell number and density. The plates were imaged every 6 h and cell culture medium and drug treatments were changed every 2 days.

### Micropattern assays

For assays in micropatterns, GFP-labelled *scrib*^*KD*^ cells with or without tetracycline pre-treatment (72 h) were plated onto circular adhesive patterns (Ø 800 μm) within a PEG cell-repellent surface^26^,^61^. The cells were flushed with culture medium 5 h after plating and the medium was replaced with phenol red-free DMEM (+10% FBS and 1% L-glutamine, Invitrogen) with or without tetracycline. Cell culture medium and drug treatments were changed every 2 days.

### PDMS-based cell compression assays

The GFP-labelled *scrib*^*KD*^ cells were plated onto a stretched flexible silicone substrate (Gel pak PF-60-X4, 150 μm thickness, Teltek), held in a custom-made chamber (GREM; http://www.jove.com/video/51193/stretching-micropatterned-cells-on-a-pdms-membrane). Before plating, the clamped membranes were coated with 25 μg ml^−1^ fibronectin/PBS (Sigma) for 1 h at 37 °C. The membranes were stretched precisely by 2 cm, which provided a 57% stretch over the resting length (unless otherwise specified). A PDMS rectangular chamber, with two compartments (6.6 × 13 mm each) was attached to the membrane with Baysilone paste (GE Bayer). Two densities (low and high) of tetracycline pre-treated or Nutlin-3 pre-treated (48 h) GFP-labelled *scrib*^*KD*^ cells were plated, one in each compartment. High-density cells were plated between 75,000 and 120,000 cells per compartment, forming a confluent monolayer; low-density cells were plated at 25,000–35,000 cells per compartment. The cells were allowed to adhere for 24 h and then the membrane was released to induce compression. The cells were fixed in 4% PFA/PBS (3 h after release for p53 staining, 1.5 h for P-p38 staining and 5 h for cleaved Caspase-3 staining) and processed for immunofluorescence. As per design, low- and high-density cells were stained and imaged from the same stretcher avoiding sample-to-sample variability. The p38 inhibitor was added one day before plating; the ROCK inhibitor was added 1 h before releasing the membrane.

### E-cadherin-blocking experiments

To block E-cadherin-dependent junctions, 20 h after the addition of tetracycline, the cells were incubated in 10 mM EDTA/PBS for 5 min, followed by a 20 min incubation in calcium-free DMEM (Invitrogen). The cells were then cultured in calcium-free DMEM (+10% FBS and 1% L-glutamine, Invitrogen), supplemented with an anti-E-cadherin-blocking antibody (1:200; ref. 57). Live-image analysis was started 2–4 h after the final media change and continued for at least 50 h.

### Immunofluorescence

For immunofluorescence, the cells were cultured on glass coverslips or on gridded dishes (μ-Dish 35 mm Grid-500, Ibidi). The cells were fixed for 10 min in 4% PFA/PBS, quenched for 10 min in 50 mM NH_4_Cl/PBS and then permeabilized for 10 min with 0.1% Triton X-100/PBS. The cells were blocked in 2% BSA, 2% FBS/PBS for 30 min. Primary and secondary antibodies were diluted in blocking solution diluted 1:1 in PBS. The primary antibodies were incubated for a minimum of 1 h at RT, followed by washes in PBS; secondary antibodies were incubated for a minimum of 30 min at RT followed by washes in PBS. Coverslips were mounted with FluorSave (Millipore). For immunostaining against phosphorylated proteins, fixing solution was supplemented with PhosSTOP (1 tablet per 10 ml, Sigma), all PBS solutions were substituted with TBS, and blocking solution was substituted with 5% BSA/TBS. For surface immunostaining, the cells were washed in ice-cold phenol red-free DMEM (Gibco) and incubated with primary antibody diluted in ice-cold phenol red-free DMEM at 4 °C for 45 min. The cells were then washed in ice-cold PBS before fixation at RT in 4% PFA/PBS for 10 min. Secondary antibody staining was then carried out as outlined previously.

### Western blotting

The cells were lysed in 1% SDS/PBS and 10–20 μg of protein was separated on a 4–12% gradient gel (Invitrogen) and transferred onto PVDF membrane for standard ECL blots (Anachem) or Immobilon FL PVDF for LI-COR blots (Millipore, 0.45 μm pore size). The membranes were blocked with 5% Marvel/0.05% Tween-20/PBS (PBST) for 1 h at RT, incubated in primary antibodies (in 2% Marvel/PBST) overnight at 4 °C, washed in PBST and incubated in secondary HRP-conjugated antibodies for ECL blots or Infrared fluorescent dyes (IRDye-800CW and IRDye-680RD) for LI-COR blots, diluted in 2% Marvel/PBST for 1 h at RT. After washes in PBST, the membranes were developed using standard ECL (GE Healthcare) or scanned with a LI-COR Odyssey CLx near-infrared fluorescence imaging system. Quantifications for LI-COR blots were carried out using Image studio lite (http://www.licor.com/bio/products/software/image_studio_lite/?gclid=COWG_Yze98MCFYvpwgodfR8Adg), using Actin to normalize the samples for loading. For original full blots, see Supplementary Fig. 9.

### RNA-seq and differential expression analysis

RNA-seq libraries were prepared with the TrueSeq RNA sample preparation V2 kit (Illumina) according to the manufacturer's instructions, and sequenced on an Illumina HiSeq 2000 instrument in single-read mode at 36 or 40 base length. The resulting fastq files were filtered for low-quality reads (<Q20) and low-quality bases were trimmed from the ends of the reads (<Q20).

Genome-based RNA-seq mapping was carried out using *Canis lupus familiaris* 3.1 (NCBI/Dog Sequencing Consortium) as a reference genome. Transcript sequences were assigned to genome using BLAT^62^. The resulting mappings were filtered by a mismatch threshold (2%), as well as requiring 90% of the transcript to match the genome and all exons to match a single chromosome. This resulted in 21,571 transcripts mapping to the genome. This mapping was used as a junction file for Tophat 2 (ref. 63), which was used to map the RNA-seq reads to the genome. To provide gene names, transcript sequences were downloaded from the NCBI RefSeq database in March 2013 (24,538 sequences). Orthologues were found against the *Mus musculus* proteome (downloaded in January 2013—NCBI RefSeq) using Inparanoid^64^. The sequences were further annotated using InterProScan^65^ to provide both InterPro Domains and Panther ontology terms^66^. For differential expression, read counts were generated by quantifying overlaps with transcript locations. These were then used to generate RPKMs. Comparisons were made between pairs of conditions, each with at least four replicates. For a transcript to be included, counts per million had to be above 10 for all samples in at least one condition and within 2-fold between replicates. Differentially expressed transcripts were then called using EdgeR^67^. Hits were selected applying the following thresholds: *P*>0.05 log FC (fold change) >0.5. Gene Ontology terms over-represented among these lists were found using David Bioinformatics Resources^68^, in particular, KEGG pathway analysis.

### Experiments in *Drosophila* wing discs

Flies were raised on a standard fly food containing yeast at 25 °C. The larvae were collected at wandering third instar stage. Clones in wing discs were induced either with en-FLP (Supplementary Fig. 5a,b) or with hs-FLP (with a 10-min heat-shock at 37 °C, 48–72 h before dissection; Supplementary Fig. 5c). For immunofluorescence, late third instar larvae were dissected in PBS followed by fixation in 4% formaldehyde/PBS solution for 20 min, permeabilization in 0.25% Triton X-100 PBS for 20 min and blocking in 4% FBS/PBS for 30 min. The primary and secondary antibodies were diluted in blocking solution. The primary antibodies were incubated overnight at 4 °C, followed by washes in 0.25% Triton X-100/PBS; secondary antibodies were incubated for a minimum of 1 h at RT followed by washes in 0.25% Triton X-100/PBS. The wing discs were mounted in Vectashield (Vector Laboratories) and imaged on Leica SP5 or SP8 confocal microscopes. Optical sections were acquired with 0.8μm steps. The images were processed in ImageJ and Photoshop. Genotypes: Supplementary Fig. 5a,b en-flp, Act>STOP>Gal4, UAS-GFP/+; UAS-Scrib-RNAi, Supplementary Fig. 5c (left) hs-FLP, tub-Gal4, UAS-GFP/+; +/+, FRT82, tub-Gal80/ FRT82, scrib2 and Supplementary Fig. 5c (right) hs-FLP, tub-Gal4, UAS-GFP/+; UAS-p53DN/+, FRT82, tub-Gal80/ FRT82, scrib2. Antibodies used: anti-Scribble (1:2000, from Chris Doe lab), anti-E-Cadherin (DSHB DCAD2, 1:200). Both the antibodies were raised against *Drosophila* antigens and have been previously used in *Drosophila*.

### Imaging and image analysis

Quantifications shown in the figures are from a single representative experiment of a minimum of three independent repeats per experiment, unless otherwise specified in the figure legend.

Fixed samples were imaged with a Leica SP5 or SP8 confocal microscope. For live imaging, the cells (kept at 37 °C and 5% CO_2_) were imaged using a Nikon BioStation CT with a × 10 air objective with imaging frequency between every 10 min and every 6 h (as indicated in the movie time stamps), with media changes every 2–3 days. For each live imaging experiment, at least five fields were imaged by time lapse and analysed. Movie sequences or individual field time points that lost focus during the experiment were discarded from further analysis, as this precluded accurate cell counting.

For Fig. 1a–c and Supplementary Fig. 1d, quantifications of cell number over time were carried out using the open-source image analysis software Cell Profiler (http://www.cellprofiler.org/), using the nuclear GFP signal to segment cells.

To measure cell density in competition assays, the number of nuclei was manually counted using DAPI and/or nuclear GFP and divided by surface area, as calculated in Fiji (http://fiji.sc/Fiji; Fig. 1g and 2n, Fig. 5e, Supplementary Fig. 1e).

For Fig. 1k–n, cell number over time was quantified with the open-source image analysis software Icy^69^, using Icy Protocols. The nuclei were detected using the wavelet-based spot detection plugins. Areas with cysts, where nuclei were not visible due to overexposure or because they were out of focus, and areas without cells were excluded from both cell counting and from area measurements on a frame by frame basis. For Fig. 3h, Fig. 4c,k and Fig. 5b, entire wells of a 96-well plate were imaged by tiling. Individual images were then stitched and processed as above.

Cell height (μm, Fig. 1h–j) was measured from apical to basal membranes using the open image analysis software Fiji.

Cell death in compression assays was quantified as the number of activated caspase-3 positive death events (Fig. 1o–q, Fig. 3i, Fig. 4d,l and Fig. 5c) divided by total number of DAPI-positive nuclei.

Kymographs were generated by first registering the hyperstack containing the different channels (bright-field, RFP and GFP) to remove any global motion or drift over time of the cells (Fig. 2d). The position of the centroid for each nucleus at each time point was extracted and the RFP and GFP channels were then projected on the same mean direction of motion by re-slicing the stacks and then projecting the maximum intensity of the centroid. For the control kymographs (GFP-labelled *scrib*^*KD*^: *scrib*^*KD*^; RFP-labelled WT: WT), this was done only using the bright-field channel and one fluorescent channel.

The directionality index of migrating cells (D=Euclidean distance/total distance, Supplementary Fig. 2b,d) was calculated after manual tracking of individual cell nuclei (>40 cells and >5 movies per condition) with the aid of ImageJ (http://imagej.nih.gov/ij/). Data analysis was performed with Matlab (MathWorks Inc.).

Analysis of cell shape and migratory features (Fig. 2g–k). The aspect ratio, which is defined as the ratio of the long axis to the short axis of cells, was measured from three independent movies by manually fitting the best fit ellipses to single cells. To plot the distribution of angles between the cell's long axis and the cell's direction of motion, we determined for each cell the orientation of the cell's long axis and calculated its angular deviation with respect to the cell's direction of motion (data were binned at 40° interval), by manual tracking. To measure the displacement of wild-type and *scrib*^*KD*^ cells, we carried out PIV analysis, using a custom algorithm based on the MatPIV software package for MATLAB, and measured the displacement of individual cells within the first row of contact. Trajectories across different movies were adjusted so that the first time point where both populations formed a broad interface and moved concertedly was set at *t*=5 h. Distance is the projected distance along the axis that joins both cell populations.

In Figs 3c–e and 4e, local density was measured for each cell by taking the sum of the Gaussian-weighted distances to all other cells within 50 μm using a Gaussian function with *σ*=20.

Nuclear p53 (Fig. 3f,g), nuclear phospho-p38 (Fig. 4a,b,i,j) and nuclear p21 (Supplementary Fig. 4d) mean intensity was measured using Volocity (http://www.perkinelmer.co.uk/pages/020/cellularimaging/products/volocity.xhtml), using DAPI as a mask to segment the nuclei.

For Fig. 5f–I, quantifications of cell number over time were carried out in ImageJ (http://imagej.nih.gov/ij/), using the nuclear Tomato signal to segment cells.

In Supplementary Fig. 2h–i, E-cadherin staining intensity was measured using Fiji. The individual cells were manually selected on an average z stack projection and their integrated density values were recorded.

### Statistical analysis

No statistical methods were used to predetermine sample size. Every experimental condition and treatment was carried out alongside a complete control set of experiments or no treatment control. The sample size was chosen to see a statistical difference between data sets. In the few instances where no difference was observed, sample size was at least as big as in conditions that had shown a difference. The experiments were not randomized and there was no blinding during experiments or analysis, as samples were marked. We carried out a minimum of independent three repeats for each experiment, unless otherwise specified in the figure legend.

The *t*-Test was used in Supplementary Fig. 1e and Supplementary Fig. 2b,d, where data were normally distributed and with equal variance. The Wicoxon rank-sum test was used in Supplementary Fig. 3f. Non-parametric Spearman correlation was used in Fig. 3e. Otherwise, the non-parametric KS test was used for all the statistical tests, removing the requirement for normally distributed data and equal variance. Throughout: *P<0.05, **P<0.005, ***P<0.0005.

## Additional information

**Accession codes:** The RNAseq data generated in this study have been deposited into the GEO (Gene Expression Omnibus) database under the accession code GSE79042.

**How to cite this article:** Wagstaff, L. *et al*. Mechanical cell competition kills cells via induction of lethal p53 levels. *Nat. Commun.* 7:11373 doi: 10.1038/ncomms11373 (2016).

## Supplementary Material

Supplementary Figures Supplementary Figures 1-9

Peer Review File Supplementary Figures 1-9

Supplementary Data 1 Genes differentially expressed upon silencing of *scribble*. List of differentially expressed genes between control cells (*scrib^KD^* -TET) and *scrib^KD^* cells +TET.

Supplementary Data 2 Genes differentially expressed between *scrib^KD^* cells that are sensitive and *scrib^KD^* cells that are resistant to out-competition. List of differentially expressed genes between *scrib^RES^* cells and *scrib^KD^* cells.

Supplementary Data 3 Intersection of the list of genes that are both differentially expressed between *scrib^KD^* and control cells and between *scrib^KD^* and *scrib^RES^* cells. List of genes that are present in both Supplementary Data 1 and 2 i.e. genes that are differentially expressed between *scrib^KD^* cells with respect to both control (*scrib^KD^* -TET) and *scrib^RES^* cells.

Supplementary Movie 1 *scrib^KD^* cells are out-competed by their wild-type neighbours but are viable as pure cultures. Left: Time-lapse of competition assay between unlabelled wild-type (WT) and GFP labelled *scrib^KD^* cells. Right: Time-lapse of co-culture of unlabelled and GFP labelled *scrib^KD^* cells.

Supplementary Movie 2 Only clones of *scrib^KD^* cells that become fully surrounded by wild-type cells are eliminated through cell competition. Time-lapse of cell competition assay between unlabelled wild-type (WT) and GFP labelled *scrib^KD^* cells. Competition is observed in surrounded *scrib^KD^* cells (white arrow), but not in cells that are only contacted (black arrow).

Supplementary Movie 3 Forcing *scrib^KD^* cells above their natural density at confluency is sufficient to induce death and live cell extrusion. Time-lapse of GFP labelled *scrib^KD^* cells growing on micropatterns (800μm ø), with (right) or without (left) the addition of tetracycline (TET). Movies show GFP labelled nuclei.

Supplementary Movie 4 Upon contact, *scrib^KD^* and wild-type cells engage in contact mediated migration. Time-lapse of co-culture of unlabelled wild-type (WT) and GFP labelled *scrib^KD^* cells. Black asterisk marks the non-migrating end of the wild-type clone; magenta asterisk marks the initial point of contact between the two populations.

Supplementary Movie 5 Homotypic cultures, of *scrib^KD^* or wild-type MDCK cells, do not engage in contact mediated migration. Time-lapse of homotypic co-cultures of MDCK cells showing absence of contact mediated migration. Left: Co-culture of unlabelled and GFP labelled *scrib^KD^* cells. Right: Co-culture of unlabelled and GFP labelled wild-type (WT) cells. Black lines mark the initial point of contact between the cell populations.

Supplementary Movie 6 Particle image velocimetry shows wild-type cells migrating towards *scrib^KD^* cells upon contact, before *scrib^KD^* cells migrate away. Time-lapse of co-culture of unlabelled wild-type (WT) and GFP labelled *scrib^KD^* cells, analysed with particle image velocimetry (PIV; shown by red arrows). Upon contact, WT cells begin migrating towards *scrib^KD^* cells before *scrib^KD^* cells start migrating away.

Supplementary Movie 7 Contact mediated migration between *scrib^KD^* and wild-type cells results in compaction and elimination of the *scrib^KD^* cells. Time-lapse of co-culture of unlabelled wild-type (WT) and GFP labelled *scrib^KD^* cells. Extended imaging time shows compaction and elimination of *scrib^KD^* cells is a result of contact mediated migration.

Supplementary Movie 8 Inhibiting E-cadherin-mediated cell contacts prevents contact mediated migration and delays the elimination of *scrib^KD^* cells. Time-lapse of competition assay between unlabelled wild-type (WT) and GFP labelled *scrib^KD^* cells. Disrupting cell junctions by calcium withdrawal and an E-cadherin blocking antibody (right) prevents contact mediated migration and delays competition compared to control (left).

Supplementary Movie 9 Reducing E-cadherin expression in *scrib^KD^* cells down to wild-type levels prevents contact mediated migration. Time-lapse of co-culture of RFP labelled wild-type (WT) cells and unlabelled *scrib^KD^* cells with E-cadherin knockdown (*scrib^KD^* E-cadKD) showing absence of contact mediated migration. Black line represents the initial point of contact between the two populations.

Supplementary Movie 10 Competition-resistant scribble cells (*scrib^RES^*) are not eliminated by wild-type cells. Time-lapse of competition assay between unlabelled wild-type (WT) and GFP labelled *scrib^RES^* cells.

Supplementary Movie 11 CRISPR knockout of p53 in *scrib^KD^* cells protects them from out-competition by wild-type cells. Time-lapse of competition assay between unlabelled wild-type (WT) and GFP labelled *scrib^KD^* cells with knockout of p53 (*scrib^KD^* *p53^-/-^*). The clone of *scrib^KD^* *p53^-/-^* cells remaining at the end of the assay is outlined in white.

Supplementary Movie 12 Inhibition of ROCK activity during competition prevents elimination of *scrib^KD^* cells. Time-lapse of competition assay between unlabelled wild-type (WT) and GFP labelled *scrib^KD^* cells in presence of the ROCK inhibitor Y27632 (30μM).

Supplementary Movie 13 Mild, sub-lethal elevation of p53 in wild-type MDCK cells is sufficient to induce loser status and activate cell competition. Left: Time-lapse of competition assay between GFP labelled wild-type (WT) cells and unlabelled WT cells with knockout of p53 (*p53^-/-^*) in presence of Nutlin-3 (8μM). Right: Homotypic culture of GFP labelled wild-type cells in presence of Nutlin-3 (8μM).

Supplementary Movie 14 Wild-type MTECs with mild, sub-lethal p53 activation are eliminated specifically in the presence of *p53^-/-^* MTECs. Left: Time-lapse of co-cultures of unlabelled mouse tracheal epithelial cells (MTECs) and Tomato labelled MTECs. Right: Time-lapse of co-cultures of unlabelled *p53^-/-^* MTECs and Tomato labelled wild-type (WT) MTEC cells. Nutlin-3 (17.5μM) was added to both co-cultures when indicated.
