# Supplementary material for: Mechanical cell competition kills cells via induction of lethal p53 levels
Source: Nat Commun. 2016 Apr 25;7:11373. doi: 10.1038/ncomms11373 (PMC4848481; doi:10.1038/ncomms11373)
Supplement: Supplementary Figures — 1-9 [file ncomms11373-s18.pdf]

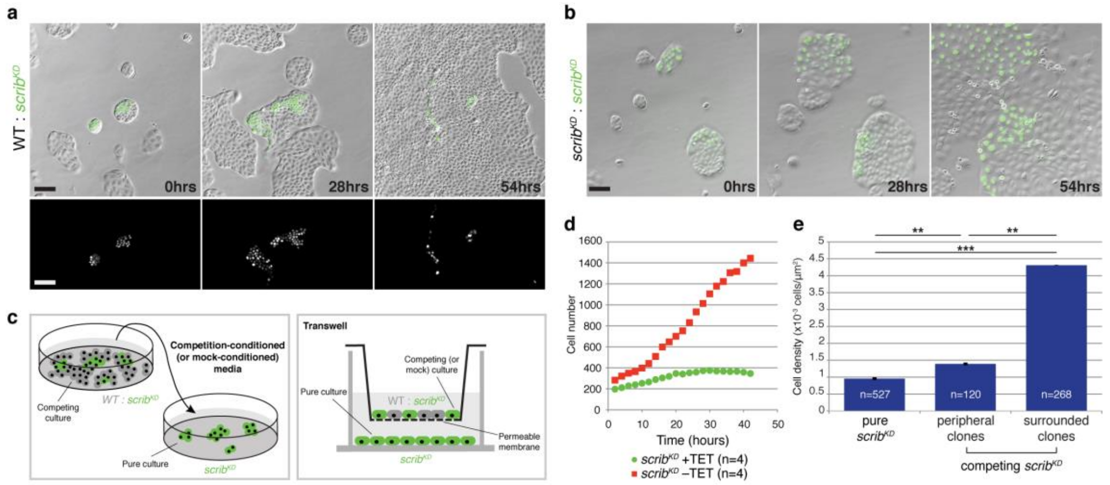

Supplementary Fig. 1. *scrib*^{KD} cells grow more slowly than wild-type cells and are compacted and eliminated when surrounded by wild-type cells.

a, b, Time-course of cell competition assays between unlabelled wild-type (WT) and GFP labelled *scrib*^{KD} cells (**a**) and mock competition between unlabelled *scrib*^{KD} and GFP labelled *scrib*^{KD} cells (**b**), see corresponding Supplementary Movie 1. Inset below (**a**) shows the GFP channel from corresponding stills. **c,** Schematic representation of the experimental set-ups for experiments with competition-conditioned or mock-conditioned medium (as in Fig. 1b) and with transwells (as in Fig. 1c). **d,** Quantification of the growth rate of *scrib*^{KD} cells with or without the addition of tetracycline (TET). Each dot represents the average of 4 fields of cells (n). **e,** Average cell density of pure confluent *scrib*^{KD} cells and of subconfluent competing *scrib*^{KD} cells that are either entirely surrounded by WT cells (surrounded clones) or merely contacting WT clones (peripheral clones). Despite being subconfluent, *scrib*^{KD} cells in competing cultures have a higher density than in confluent pure cultures; n = cell number, mean ± sem. Scale bars: movie sequences = 100μm and immunofluorescence images = 50μm here and throughout all Supplementary Figures. ** $p<0.005$, *** $p<0.0005$ by T-test.

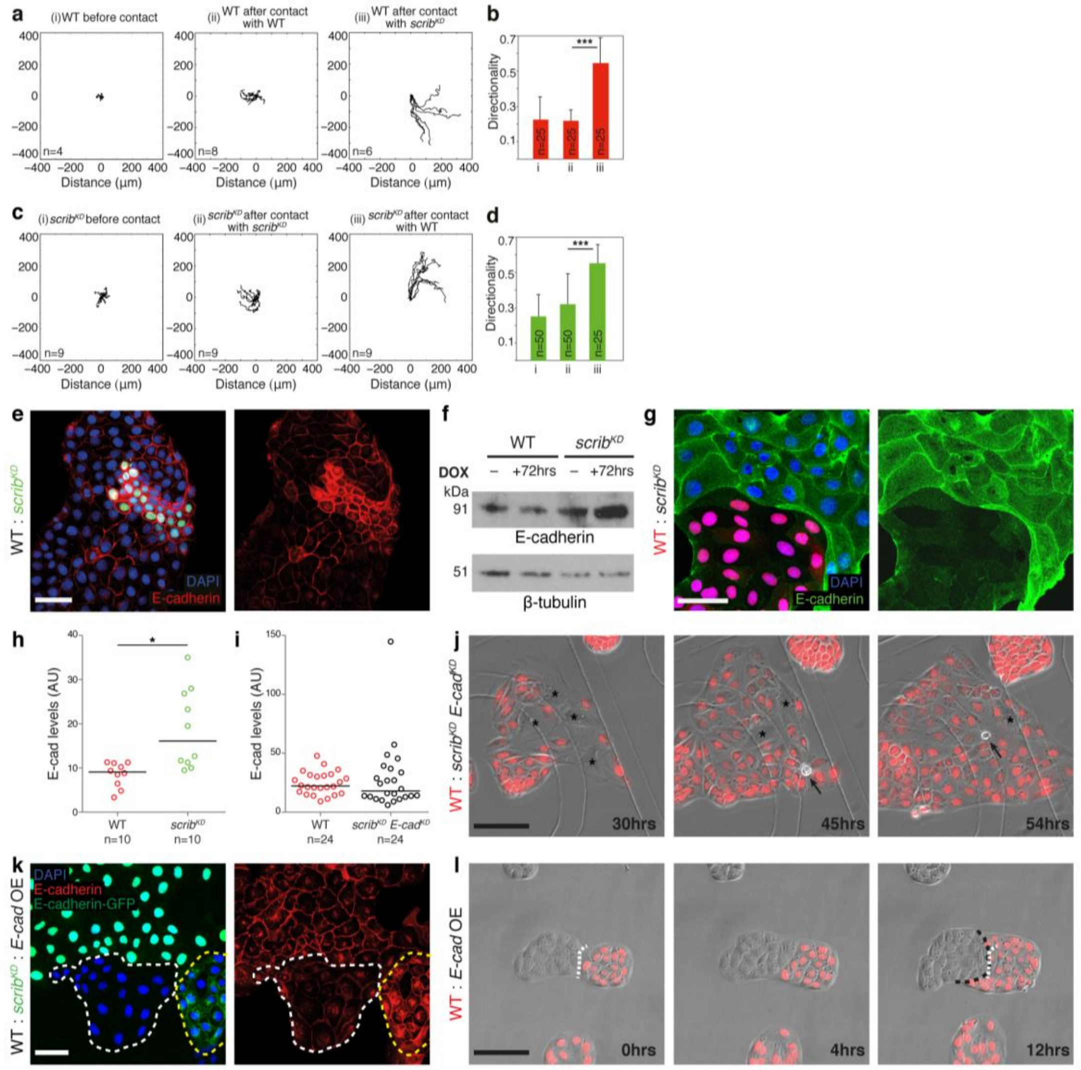

Supplementary Fig. 2. E-cadherin is upregulated in *scrib*^{KD} cells and E-cadherin upregulation is not sufficient to induce directional migration.

a-d, Individual cell trajectories of wild-type (WT) (**a**) or *scrib*^{KD} (**c**) cells before or after contact as indicated, and corresponding average directionalities. **(b, d)**. Error bars = SD; quantifications from pooled data from 3 biological replicates; *** $p<0.0005$ by T-test. **e,** Anti-E-cadherin staining of co-cultures of WT and GFP labelled *scrib*^{KD} cells shows accumulation of E-cadherin in *scrib*^{KD} cells compared to WT cells (sum intensity projection). **f,** Western blot against E-cadherin in WT and *scrib*^{KD} cells +/- doxycycline (DOX). β-tubulin was used as loading control. **g,** Cell surface E-cadherin staining of RFP labelled WT and unlabelled *scrib*^{KD} co-cultures shows surface accumulation of E-cadherin in *scrib*^{KD} cells compared to WT cells. **h, i,** Quantification of single cell E-cadherin intensities from one representative set of confocal images as in Fig. 2q, rightmost panel; black bars = median; * $p<0.05$ by KS test. **j,** Stills from time-lapse movie of RFP labelled WT and unlabelled *scrib*^{KD} *E-cad*^{KD} co-cultures showing that the latter are still eliminated by WT cells despite lack of contact-induced migration. Asterisks mark individual *scrib*^{KD} *E-cad*^{KD} cells; black arrows = cell death events. **k,** Immunofluorescence staining comparing E-cadherin levels between WT cells (within white dashed line), *scrib*^{KD} cells with nuclear GFP, and cells overexpressing GFP labelled E-cadherin (*E-cad* OE; within yellow dashed

line). **l,** Co-culture of RFP labelled WT cells and *E-cad* OE cells shows no directional migration upon contact (white dashed line = initial point of contact; black dashed line = final point of contact). n = number of cells.

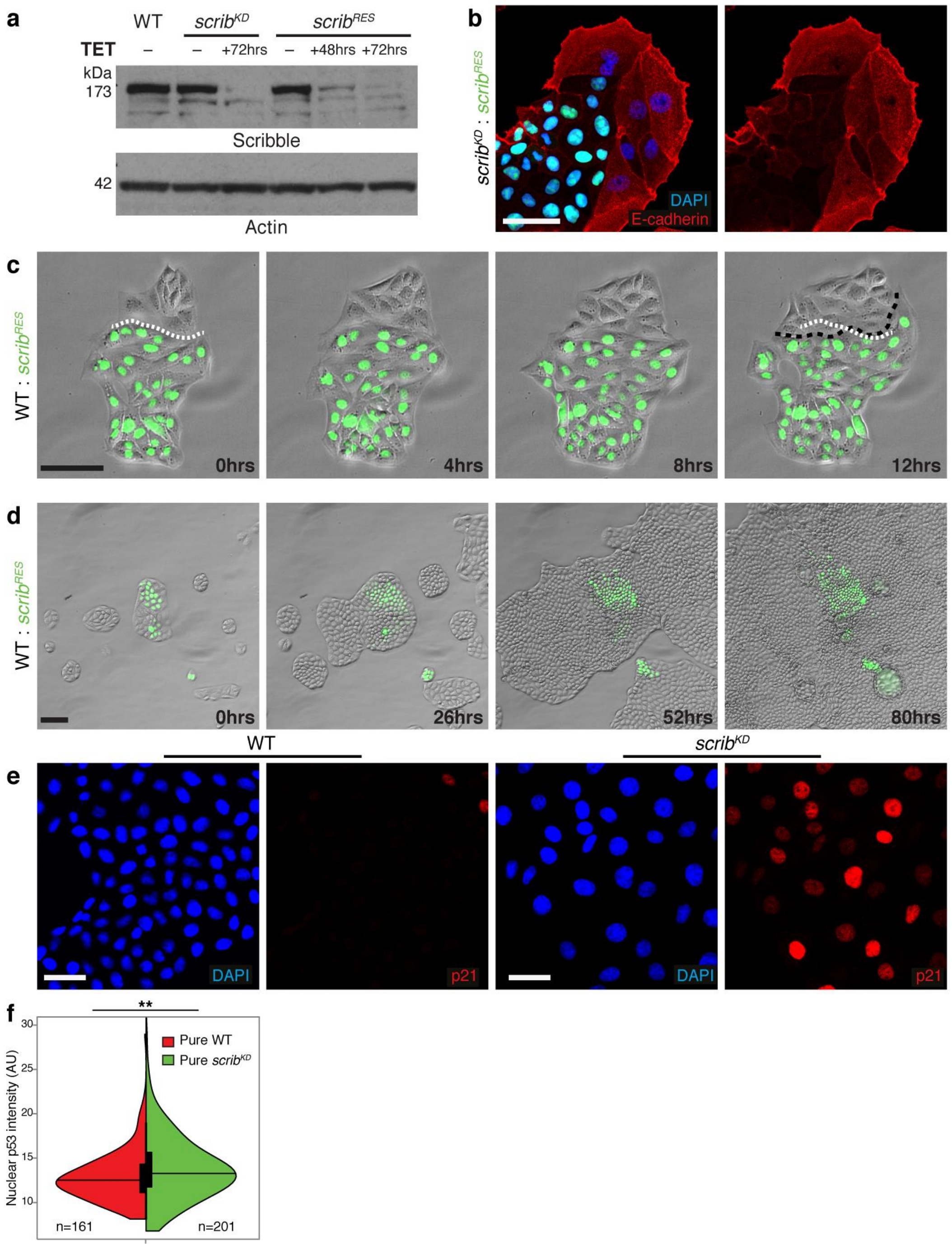

Supplementary Fig. 3. Characterisation of *scrib*^{RES} cells and analysis of p21 and p53 expression in *scrib*^{KD} cells.

a, Western blot against Scribble for *scrib*^{KD} and *scrib*^{RES} cells +/- TET. **b,** Surface E-cadherin staining of GFP labelled *scrib*^{RES} cells mixed with unlabelled *scrib*^{KD} cells. **c, d,** Stills from time-lapse movies of wild-type (WT) and GFP labelled *scrib*^{RES} cells to assess directional migration (**c**) and competition (**d**), see corresponding Supplementary Movie 10. White dashed line = initial point of contact; black dashed line = final point of contact. **e,** p21 staining of pure WT cells and pure *scrib*^{KD} cells. Note widespread p21 elevation in *scrib*^{KD} cells. **f,** Loss of Scribble leads to an overall

increase in nuclear p53 staining. n = cell number; ** $p<0.005$ by Wicoxon rank sum test.

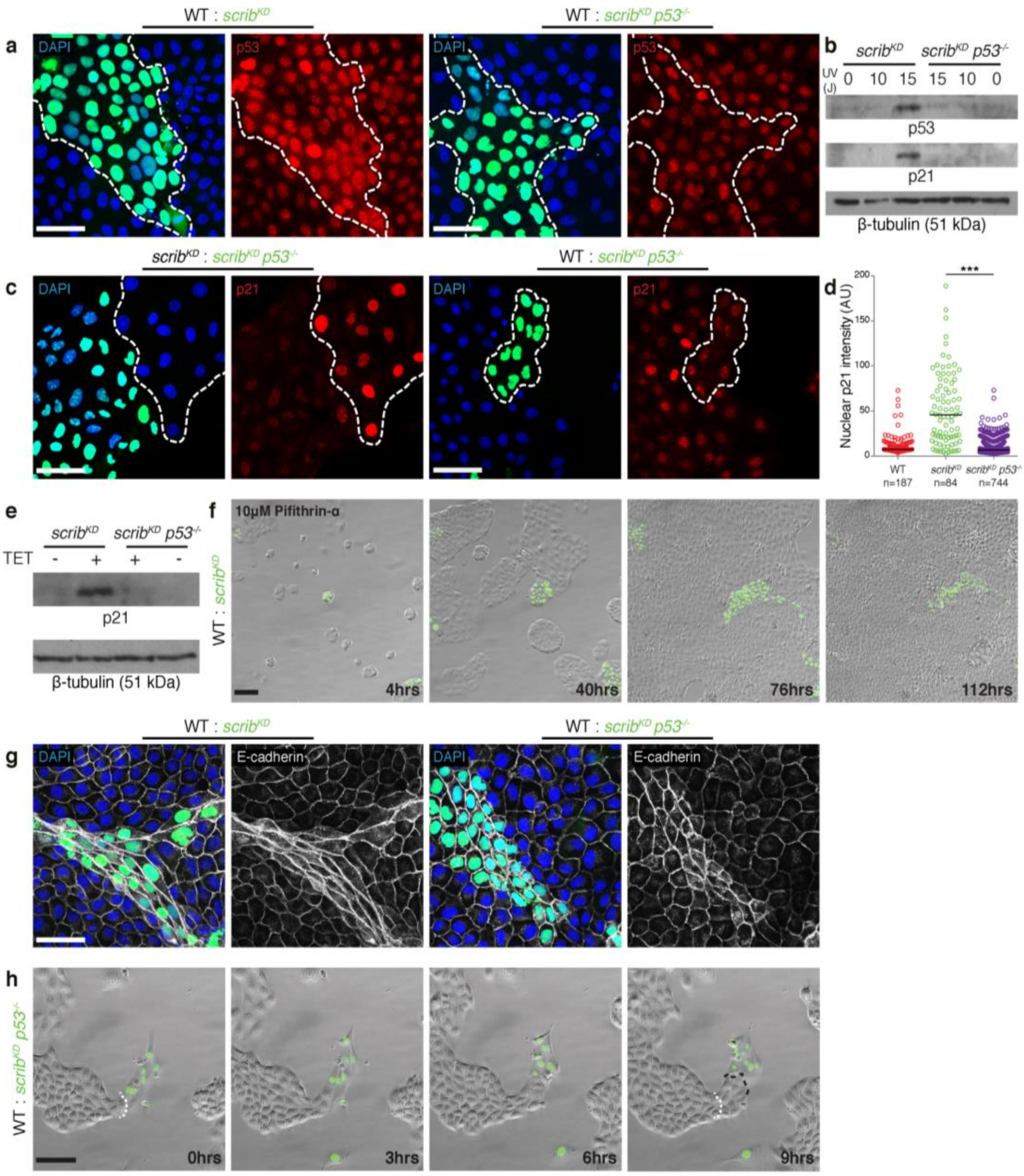

Supplementary Fig. 4. Loss of p53 activity blocks elimination of *scrib*^{KD} cells, but does not prevent up-regulation of E-cadherin or contact-induced migration.

a, Anti-p53 staining of GFP labelled *scrib*^{KD} or GFP labelled *scrib*^{KD} *p53*$^{-/-}$ cells in competition with wild-type (WT) cells. Two fields of similar cell densities are shown for comparison. **b,** Western blot against p53 and p21 in *scrib*^{KD} and *scrib*^{KD} *p53*$^{-/-}$ cells +/- UV-C irradiation; β-tubulin as loading control. **c,** Anti-p21 staining of *scrib*^{KD} *p53*$^{-/-}$ cells when cultured next to *scrib*^{KD} or WT cells (all images are taken from the same coverslip using the fence system). **d,** Single cell nuclear p21 intensity from confocal images as in (**c**); *** $p<0.0005$ by KS test. **e,** Western blot against p21 in *scrib*^{KD} and *scrib*^{KD} *p53*$^{-/-}$ cells +/- TET; β-tubulin as loading control. **f,** Stills from time-lapse movies of WT and GFP labelled *scrib*^{KD} competition assays in the presence of Pifithrin-α (10μM). **g,** Anti-E-cadherin staining of GFP labelled *scrib*^{KD} or GFP labelled *scrib*^{KD} *p53*$^{-/-}$ cells co-cultured with WT cells. **h,** Stills from time-lapse movies with WT and GFP labelled *scrib*^{KD} *p53*$^{-/-}$ cells. n = cell number. 2 independent repeats per experiment for (**a, b, d**).

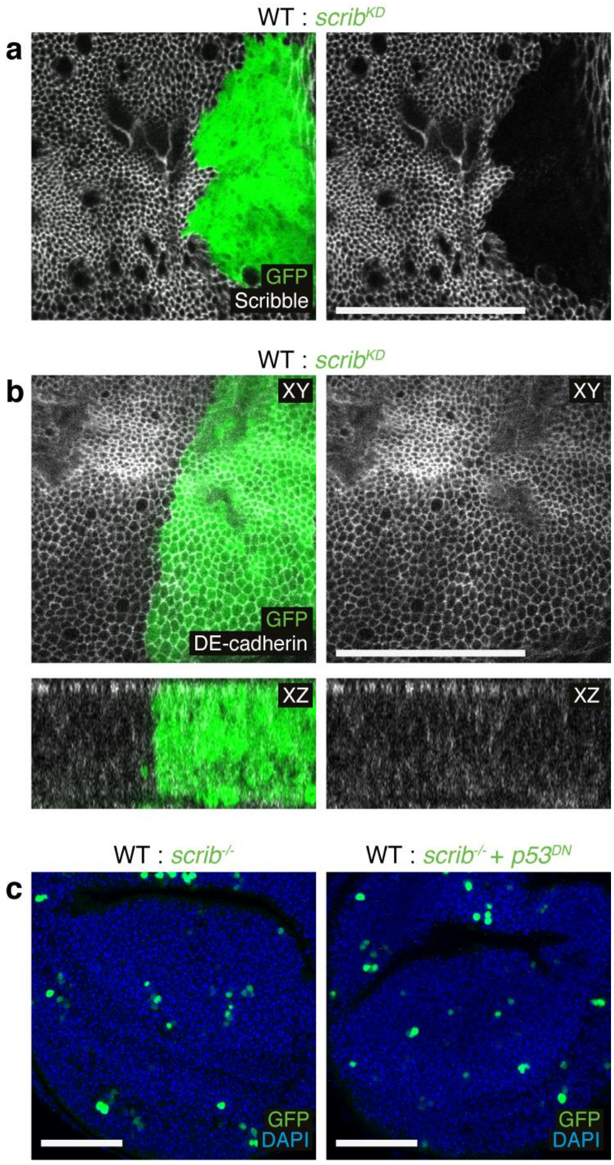

Supplementary Fig. 5. ***scrib*** $^{-/-}$ **clones are eliminated in a p53-independent manner in *Drosophila* wing discs.**

a, Scribble immunostaining of *Drosophila* wing disc with GFP labelled clones expressing Scribble RNAi; 1 experimental repeat; n = 6 wing discs. The same result was obtained with several independent drivers. **b,** XY and XZ views of DE-cadherin staining of *Drosophila* wing disc with GFP labelled Scribble RNAi clones. XY view shows average projection of multiple z-sections acquired at the plane of the adherens junctions; 1 experimental repeat; n = 4 wing discs. The same result was obtained with several independent drivers. **c,** Representative images of 48 hour old GFP labelled *scrib* $^{-/-}$ clones (control) or *scrib* $^{-/-}$ clones overexpressing $p53^{DN}$; 2 experimental repeats; n > 3 wing discs per condition/experiment. The same result was obtained with a validated p53RNAi line.

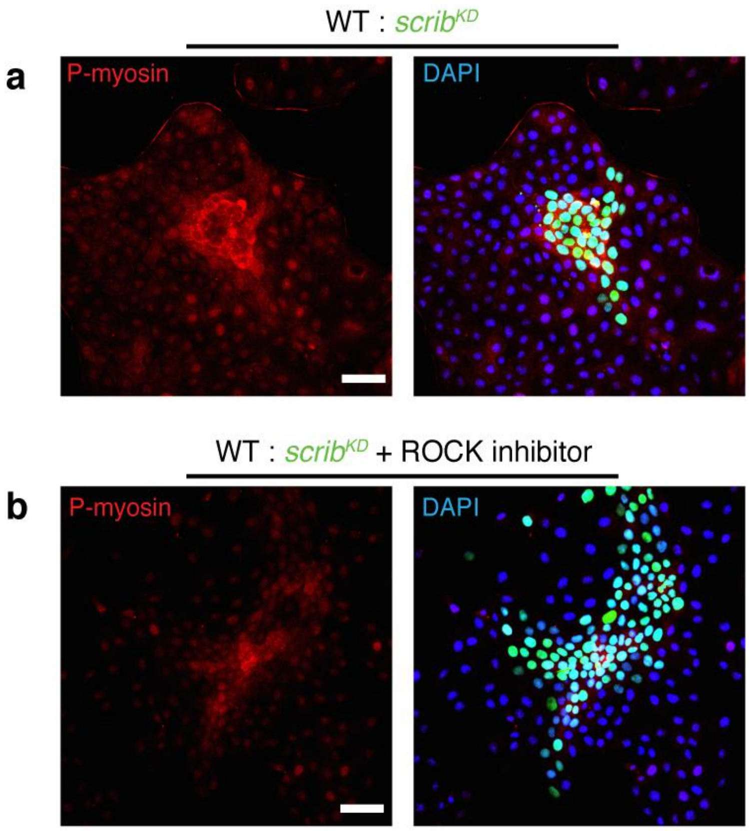

Supplementary Fig. 6. ROCK is required for P-myosin increase.
a, b, Active P-myosin II (phospho S20) immunofluorescence staining of GFP labelled *scrib*^{KD} and unlabelled wild-type (WT) co-cultures without (**a**) or with (**b**) addition of ROCK inhibitor (Y27632).

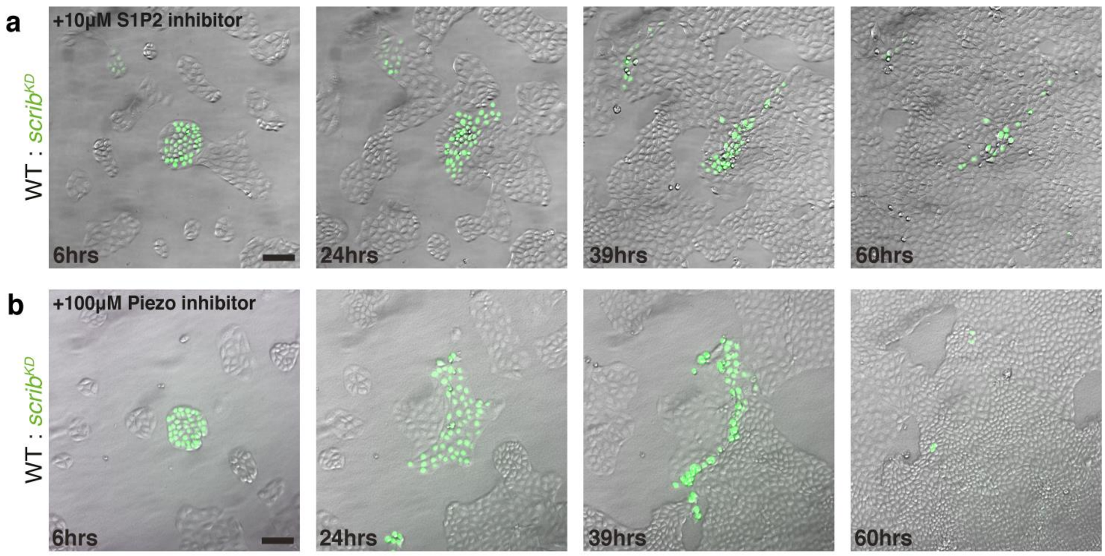

Supplementary Fig. 7 Neither S1P2 nor Piezo are required for *scrib*^{KD} cell elimination.

a, b, Stills from time-lapse movies of WT and *scrib*^{KD} co-cultures in the presence of a S1P2 inhibitor (JTE013) (**a**) or Piezo inhibitor (gadolinium III chloride) (**b**).

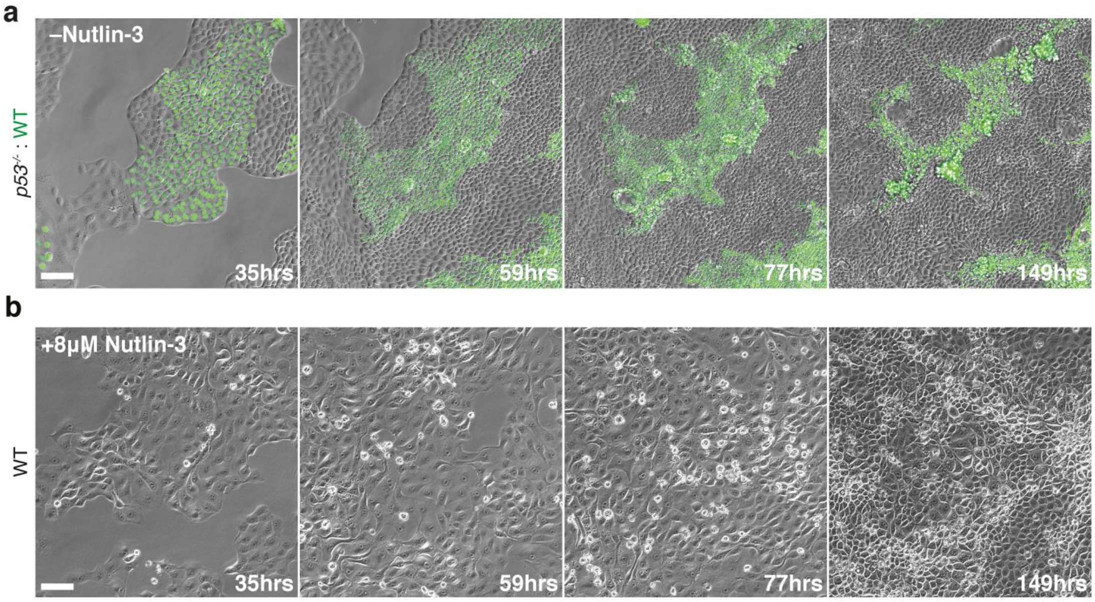

Supplementary Fig. 8. p53 activation is sufficient to cause flattening of wild-type MDCK cells.

a, Stills from time-lapse movies of co-cultures of GFP labelled wild-type (WT) and unlabelled *p53*$^{-/-}$ MDCK cells. **b,** Stills from time-lapse movies of pure WT MDCK cells with Nutlin-3 (8μM, see Supplementary Movie 13 right).

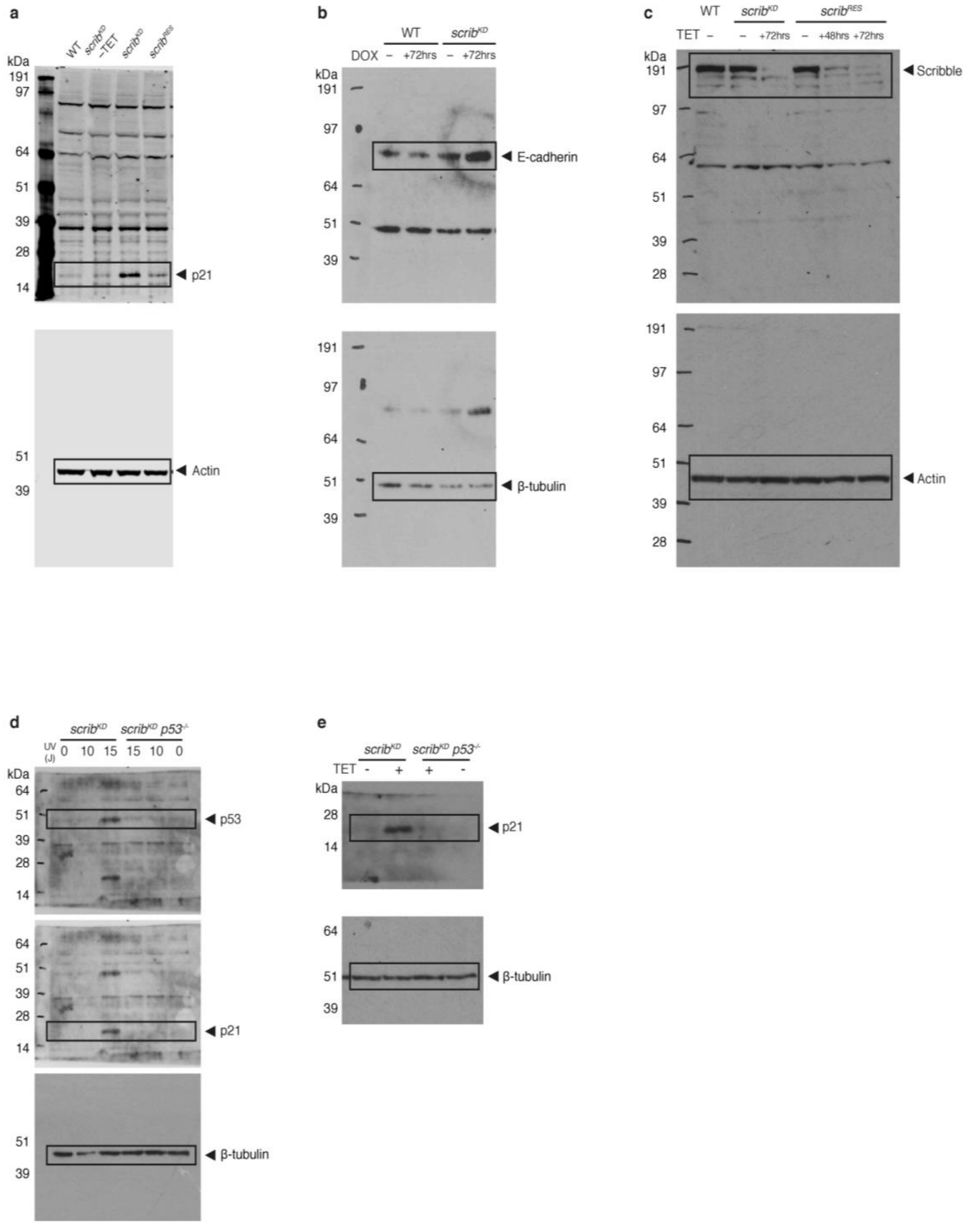

Supplementary Fig. 9. Un-cropped original Western blots.
a, Western blots showing p21 and loading control actin from Fig. 3b. **b,** Western blots showing E-cadherin and loading control β-tubulin from Supplementary Fig. 2f. **c,** Western blots showing Scribble and loading control actin from Supplementary Fig. 3a. **d,** Western blots showing p53, p21 and loading control β-tubulin from Supplementary Fig. 4b. **e,** Western blots showing p21 and loading control β-tubulin from Supplementary Fig. 4e. Black box represents the area of each blot used in each Figure.
