# Supplementary material for: Mechanical cell competition kills cells via induction of lethal p53 levels
Source: Nat Commun. 2016 Apr 25;7:11373. doi: 10.1038/ncomms11373 (PMC4848481; doi:10.1038/ncomms11373)
Supplement: Peer Review File — Supplementary Figures 1-9 [file ncomms11373-s19.pdf]

Editorial Note: this manuscript has been previously reviewed at another journal that is not operating a transparent peer review scheme. This document only contains reviewer comments and rebuttal letters for versions considered at Nature Communications.
